# Supplementary material for: Phylogenetic Analysis and Codon Usage Bias Reveal the Base of Feline and Canine Chaphamaparvovirus for Cross-Species Transmission
Source: Animals (Basel). 2023 Aug 14;13(16):2617. doi: 10.3390/ani13162617 (PMC10451695; doi:10.3390/ani13162617)
Supplement: Supplementary file 1 [file animals-13-02617-s001.zip › Supplementary Table 2-2.pdf]

Genome

Percent Identity

Divergence

	1	2	3	4	5	6	7	8	9	10	11	12	13	14	15	16	17	18	19	20	21	22	23	24	25	26	27	28	29	30		
1		99.8	98.8	98.6	98.9	98.8	98.9	99.4	99.3	98.7	98.9	99.1	99.3	94.0	98.9	94.3	73.3	73.1	74.0	74.0	74.0	74.1	71.0	70.9	70.7	71.1	74.2	74.1	74.1	73.7	1	MH893826.1-Cachavirus-Canine-2017-USA
2	0.2		98.6	98.6	99.0	98.7	98.9	99.2	99.2	98.8	98.9	99.1	99.4	94.0	98.9	94.2	73.3	73.0	73.9	73.9	73.9	74.0	70.3	70.2	69.3	70.0	74.1	74.0	74.0	73.6	2	MK448316.1-Cachavirus-Canine-2018-USA
3	1.3	1.3		98.8	98.4	98.7	98.7	99.0	99.0	98.7	98.6	98.8	99.0	93.4	98.5	93.5	73.5	73.4	73.5	73.5	73.5	73.6	72.5	72.6	72.4	72.7	73.7	73.6	73.6	73.2	3	MT123283.1-Chapparvovirus-Canine-2018-C
4	1.4	1.3	1.2		98.6	98.7	99.0	98.5	98.5	98.9	99.1	98.7	99.0	93.4	98.6	93.3	73.6	73.5	73.6	73.6	73.6	73.6	72.6	72.7	72.5	72.8	73.8	73.6	73.7	73.3	4	MT123284.1-Chapparvovirus-Canine-2019-C
5	1.1	0.9	1.6	1.4		98.8	98.9	98.8	98.8	98.8	98.8	98.9	99.1	93.4	98.8	93.5	73.7	73.6	73.7	73.6	73.6	73.7	72.7	72.7	72.5	72.8	73.9	73.7	73.8	73.4	5	MT123285.1-Chapparvovirus-Canine-2019-C
6	1.2	1.3	1.3	1.4	1.2		99.0	98.7	98.7	98.8	98.9	98.8	99.0	93.5	98.6	93.4	73.7	73.6	73.7	73.7	73.7	73.8	72.7	72.8	72.6	72.9	73.9	73.8	73.8	73.4	6	MT123286.1-Chapparvovirus-Canine-2019-C
7	1.1	1.1	1.3	1.1	1.1	1.0		98.7	98.7	99.1	99.2	98.9	99.1	93.5	98.7	93.4	73.6	73.5	73.6	73.6	73.6	73.6	72.6	72.7	72.5	72.8	73.9	73.6	73.7	73.3	7	MT123287.1-Chapparvovirus-Canine-2019-C
8	0.6	0.7	1.0	1.5	1.2	1.3	1.3		99.9	98.8	98.7	99.2	99.3	94.2	98.9	94.3	73.2	73.1	74.0	74.0	74.0	74.0	72.1	72.1	71.9	72.2	74.2	74.0	74.0	73.7	8	MT710947.1-Carnivore_chaphamaparvovirus
9	0.7	0.7	1.1	1.5	1.2	1.3	1.3	0.1		98.8	98.6	99.2	99.3	94.2	98.9	94.4	73.2	73.1	73.9	73.9	73.9	74.0	72.0	72.0	71.8	72.2	74.1	74.0	74.0	73.6	9	MT710948.1-Carnivore_chaphamaparvovirus
10	1.3	1.2	1.3	1.1	1.2	1.2	0.9	1.2	1.2		99.1	99.0	99.2	93.6	98.8	93.5	73.7	73.6	73.7	73.7	73.7	73.8	72.8	72.8	72.6	72.9	74.0	73.8	73.8	73.4	10	MN928790.1-Chapparvovirus_sp-Feline-201
11	1.1	1.1	1.4	0.9	1.2	1.1	0.8	1.4	1.4	0.9		98.8	99.1	93.5	98.7	93.4	73.7	73.6	73.7	73.7	73.7	73.8	72.7	72.7	72.6	72.9	73.9	73.7	73.8	73.4	11	MN928791.1-Chapparvovirus_sp-Feline-201
12	0.9	0.8	1.1	1.3	1.1	1.2	1.1	0.8	0.8	1.0	1.1		99.6	94.0	99.0	94.2	73.2	73.0	73.9	73.9	73.9	74.0	71.2	71.2	71.0	71.4	74.1	74.0	74.0	73.6	12	OK546100.1-Carnivore_chaphamaparvovirus
13	0.7	0.6	1.0	1.0	0.9	1.0	0.9	0.7	0.7	0.8	0.9	0.4		94.0	99.3	94.2	73.3	73.1	74.0	74.0	74.0	74.0	71.2	71.2	71.1	71.4	74.2	74.0	74.1	73.7	13	OK546101.1-Carnivore_chaphamaparvovirus
14	6.2	6.2	6.9	7.0	6.9	6.8	6.9	6.1	6.0	6.7	6.9	6.2	6.2		94.0	97.8	73.0	72.7	73.5	73.5	73.5	73.6	72.3	72.4	72.5	72.6	73.7	73.6	73.8	73.3	14	OK546102.1-Carnivore_chaphamaparvovirus
15	1.0	0.9	1.3	1.3	1.1	1.3	1.2	1.0	1.0	1.1	1.2	0.8	0.5	6.2		94.0	73.1	72.9	73.7	73.7	73.7	73.7	72.2	72.2	72.0	72.4	73.9	73.7	73.8	73.4	15	OM640108.1-Carnivore_chaphamaparvovirus
16	6.0	6.0	6.9	7.1	6.8	6.9	7.0	5.9	5.9	6.8	6.9	6.1	6.0	2.2	6.1		73.2	72.9	73.8	73.8	73.8	73.8	71.2	71.3	71.4	71.5	73.8	73.8	73.9	73.5	16	OM640109.1-Carnivore_chaphamaparvovirus
17	33.0	33.0	32.7	32.6	32.4	32.4	32.6	33.2	33.2	32.4	32.4	33.1	33.0	33.5	33.2	33.3		98.6	99.3	99.3	99.3	99.3	98.6	98.7	98.5	98.5	98.7	98.8	99.2	98.2	17	MZ031965.1-Feline_chaphamaparvovirus-Fe
18	33.4	33.4	32.8	32.7	32.6	32.6	32.7	33.3	33.4	32.5	32.6	33.4	33.3	33.9	33.5	33.7	1.4		98.5	98.5	98.4	98.5	98.1	98.9	99.3	99.4	99.4	98.9	98.7	99.4	18	MZ031966.1-Feline_chaphamaparvovirus-Fe
19	31.9	32.0	32.7	32.5	32.4	32.4	32.5	32.0	32.0	32.4	32.4	32.0	31.9	32.6	32.2	32.3	0.7	1.5		99.9	99.9	100.0	98.8	98.8	98.6	98.4	98.5	98.6	99.2	98.0	19	OP499830.1-Feline_chaphamaparvovirus-Fe
20	31.9	32.0	32.7	32.6	32.5	32.4	32.6	32.0	32.1	32.4	32.4	32.1	32.0	32.7	32.3	32.3	0.7	1.6	0.1		99.9	99.9	98.7	98.8	98.5	98.3	98.4	98.5	99.3	97.9	20	OP499831.1-Feline_chaphamaparvovirus-Fe
21	31.9	32.0	32.7	32.6	32.5	32.4	32.6	32.0	32.1	32.4	32.4	32.1	32.0	32.7	32.3	32.3	0.7	1.6	0.1	0.1		99.9	98.7	98.7	98.5	98.3	98.3	98.5	99.2	97.9	21	OP499832.1-Feline_chaphamaparvovirus-Fe
22	31.9	31.9	32.6	32.5	32.4	32.3	32.5	31.9	32.0	32.3	32.3	32.0	31.9	32.6	32.2	32.2	0.7	1.5	0.0	0.1	0.1		98.8	98.8	98.5	98.4	98.4	98.6	99.3	97.9	22	OP499833.1-Feline_chaphamaparvovirus-Fe
23	36.7	37.8	34.3	34.1	34.0	34.0	34.1	35.0	35.1	33.8	34.0	36.4	36.3	34.6	34.6	36.3	1.4	1.9	1.2	1.3	1.4	1.3		99.0	98.1	98.0	98.2	98.1	98.5	97.7	23	OQ162042.1-Feline_chaphamaparvovirus-Ca
24	36.9	38.0	34.2	34.0	33.9	33.9	34.0	35.0	35.1	33.8	33.9	36.4	36.3	34.5	34.6	36.3	1.3	1.1	1.2	1.3	1.3	1.2	1.0		99.1	98.9	99.0	98.6	98.5	98.4	24	OQ162043.1-Feline_chaphamaparvovirus-Ca
25	37.1	39.4	34.4	34.2	34.2	34.1	34.2	35.2	35.3	34.2	34.1	36.6	36.5	34.2	34.9	35.9	1.4	0.7	1.4	1.5	1.5	1.4	1.9	0.9		98.5	99.4	98.8	98.5	98.8	25	MN396757.1-Feline_chaphamaparvovirus-Fe
26	36.6	38.3	34.0	33.8	33.7	33.7	33.8	34.7	34.8	33.6	33.7	36.1	36.0	34.1	34.3	35.9	1.6	0.6	1.6	1.7	1.7	1.6	2.0	1.2	1.5		99.2	98.7	98.5	99.0	26	MN794869.1-Carnivore_chapparvovirus_1-F
27	31.7	31.7	32.4	32.2	32.1	32.0	32.1	31.6	31.7	32.0	32.1	31.7	31.6	32.4	31.9	32.2	1.3	0.6	1.6	1.6	1.7	1.6	1.9	1.0	0.6	0.8		98.8	98.3	98.8	27	MW404252.1-Feline_chaphamaparvovirus-Fe
28	31.9	31.9	32.6	32.5	32.4	32.3	32.5	31.9	32.0	32.3	32.4	32.0	31.9	32.5	32.2	32.3	1.2	1.1	1.4	1.5	1.5	1.4	2.0	1.4	1.1	1.3	1.2		98.5	98.3	28	MW404253.1-Feline_chaphamaparvovirus-Fe
29	31.8	31.8	32.5	32.4	32.3	32.2	32.4	31.9	31.9	32.2	32.2	31.9	31.8	32.3	32.1	32.1	0.8	1.4	0.8	0.7	0.8	0.7	1.5	1.5	1.5	1.6	1.7	1.5		98.1	29	MT708230.1-Feline_chaphamaparvovirus-Fe
30	32.4	32.5	33.1	33.0	32.9	32.8	33.0	32.4	32.5	32.8	32.9	32.5	32.4	33.0	32.6	32.8	1.9	0.6	2.1	2.2	2.2	2.1	2.4	1.6	1.2	1.0	1.2	1.7	1.9		30	MT708231.1-Feline_chaphamaparvovirus-Fe
	1	2	3	4	5	6	7	8	9	10	11	12	13	14	15	16	17	18	19	20	21	22	23	24	25	26	27	28	29	30		

NS nucleotide

Percent Identity

Divergence

	1	2	3	4	5	6	7	8	9	10	11	12	13	14	15	16	17	18	19	20	21	22	23	24	25	26	27	28	29	30		
1		99.2	99.3	99.3	98.5	98.3	98.3	98.6	99.3	98.5	98.4	98.4	98.5	99.0	98.2	75.0	74.6	74.6	74.7	74.7	74.6	75.1	75.0	74.8	74.7	74.9	75.1	74.5	74.8	74.9	1	Feline-chaphamaparvovirus-MN396757.1-Ca
2	0.8		99.1	99.3	98.4	98.4	98.6	98.6	99.6	98.5	98.4	98.4	98.5	99.0	98.2	75.0	74.5	74.5	74.7	74.6	74.5	75.1	75.0	74.8	74.6	74.9	75.1	74.4	74.8	74.8	2	Feline-chaphamaparvovirus-MN794869.1-US
3	0.7	0.9		99.3	98.5	98.2	98.2	98.5	99.2	98.4	98.3	98.3	98.4	98.9	98.1	75.1	74.6	74.5	74.8	74.7	74.5	75.1	75.1	74.9	74.7	75.1	75.1	74.5	74.9	74.8	3	Feline_chaphamaparvovirus-MW404251.1-It
4	0.7	0.7	0.7		98.4	98.3	98.4	98.6	99.4	98.5	98.3	98.4	98.4	99.0	98.2	75.0	74.5	74.5	74.7	74.6	74.5	75.1	75.0	74.8	74.6	74.9	75.1	74.2	74.8	74.6	4	Feline_chaphamaparvovirus-MW404252.1-It
5	1.5	1.6	1.5	1.6		98.7	97.5	99.1	98.5	98.8	98.7	98.8	98.8	98.5	98.2	74.9	74.4	74.3	74.6	74.5	74.3	74.9	74.8	74.7	74.5	74.7	74.9	74.4	74.7	74.8	5	Feline_chaphamaparvovirus-MW404253.1-It
6	1.7	1.6	1.8	1.7	1.3		97.5	99.4	98.5	99.6	99.6	99.7	99.6	98.6	98.8	75.2	74.6	74.5	74.8	74.7	74.5	75.1	75.1	74.9	74.8	74.9	75.1	74.7	74.9	75.0	6	Feline_chaphamaparvovirus-MT708230.1-Ch
7	1.7	1.4	1.8	1.6	2.5	2.6		97.7	99.0	97.6	97.4	97.5	97.5	98.1	97.3	74.1	73.6	73.6	73.8	73.7	73.6	74.1	74.1	73.9	73.7	74.0	74.1	73.6	74.0	74.0	7	Feline_chaphamaparvovirus-MT708231.1-Ch
8	1.4	1.4	1.5	1.4	0.9	0.6	2.4		98.7	99.4	99.4	99.5	99.4	98.7	98.7	74.9	74.4	74.3	74.6	74.5	74.3	74.8	74.7	74.7	74.6	74.7	74.9	74.4	74.7	74.8	8	Feline_chaphamaparvovirus-MZ031965.1-Ch
9	0.7	0.4	0.8	0.6	1.5	1.5	1.0	1.3		98.6	98.4	98.5	98.5	99.1	98.3	74.8	74.4	74.3	74.6	74.5	74.3	74.9	74.8	74.7	74.5	74.7	74.9	74.2	74.7	74.6	9	Feline_chaphamaparvovirus-MZ031966.1-Ch
10	1.5	1.5	1.6	1.5	1.2	0.4	2.5	0.6	1.4		99.8	99.9	99.9	98.8	99.0	75.1	74.5	74.5	74.7	74.6	74.5	75.1	75.0	74.8	74.7	74.9	75.1	74.4	74.8	74.9	10	Feline_chaphamaparvovirus-OP499830.1-Ch
11	1.6	1.6	1.7	1.7	1.3	0.4	2.6	0.6	1.6	0.2		99.9	99.9	98.6	98.8	75.1	74.5	74.4	74.7	74.6	74.4	75.0	74.9	74.8	74.7	74.8	75.0	74.4	74.8	74.9	11	Feline_chaphamaparvovirus-OP499831.1-Ch
12	1.6	1.6	1.7	1.6	1.2	0.3	2.6	0.5	1.5	0.1	0.1		99.9	98.7	98.9	75.1	74.5	74.5	74.7	74.6	74.5	75.1	75.0	74.8	74.7	74.9	75.1	74.4	74.8	74.9	12	Feline_chaphamaparvovirus-OP499832.1-Ch
13	1.5	1.5	1.6	1.6	1.2	0.4	2.5	0.6	1.5	0.1	0.1	0.1		98.7	98.9	75.2	74.6	74.5	74.8	74.7	74.5	75.1	75.1	74.9	74.8	74.9	75.1	74.5	74.9	75.0	13	Feline_chaphamaparvovirus-OP499833.1-Ch
14	1.0	1.0	1.1	1.0	1.5	1.4	2.0	1.3	0.9	1.2	1.4	1.3	1.3		99.2	74.8	74.4	74.3	74.6	74.5	74.3	74.9	74.8	74.8	74.5	74.7	74.9	74.3	74.7	74.7	14	Feline_chaphamaparvovirus-OQ162043.1-20
15	1.8	1.8	1.9	1.9	1.9	1.2	2.8	1.3	1.7	1.0	1.2	1.1	1.1	0.8		74.9	74.3	74.3	74.5	74.4	74.3	74.8	74.8	74.7	74.4	74.7	74.8	74.2	74.6	74.7	15	Feline_chaphamaparvovirus-OQ162042.1-20
16	30.4	30.4	30.4	30.4	30.5	30.2	31.8	30.5	30.7	30.3	30.4	30.3	30.2	30.7	30.6		98.5	98.3	98.9	98.7	98.7	99.2	99.1	98.8	98.8	99.0	99.2	94.5	98.8	94.8	16	Carnivore_chapparvovirus-MK448316.1-USA
17	31.0	31.1	31.1	31.1	31.4	31.1	32.5	31.4	31.4	31.1	31.2	31.1	31.1	31.4	31.4	1.5		99.2	98.4	98.8	98.8	98.7	98.6	99.2	99.0	98.9	99.1	94.1	98.7	94.3	17	Carnivore_chapparvovirus-MT123283.1-Chi
18	31.1	31.2	31.1	31.2	31.4	31.1	32.6	31.5	31.5	31.2	31.3	31.2	31.1	31.5	31.5	1.7	0.8		98.2	98.6	98.7	98.5	98.4	98.9	98.7	98.7	98.9	94.0	98.4	94.2	18	Carnivore_chapparvovirus-MT123284.1-Chi
19	30.8	30.8	30.7	30.8	31.0	30.7	32.2	31.0	31.1	30.8	30.9	30.8	30.7	31.1	31.1	1.1	1.6	1.8		98.8	98.6	98.9	98.8	98.8	98.7	98.9	99.1	94.3	98.7	94.7	19	Carnivore_chapparvovirus-MT123285.1-Chi
20	30.8	31.0	30.9	31.0	31.2	30.9	32.4	31.2	31.2	31.0	31.0	31.0	30.9	31.2	31.3	1.3	1.2	1.4	1.2		99.1	98.6	98.5	99.0	99.1	98.8	99.0	94.3	98.6	94.4	20	Carnivore_chapparvovirus-MT123286.1-Chi
21	31.1	31.2	31.1	31.2	31.4	31.1	32.6	31.4	31.4	31.2	31.3	31.2	31.1	31.4	31.5	1.3	1.2	1.3	1.4	0.9		98.6	98.5	99.0	99.1	98.8	99.0	94.0	98.5	94.2	21	Carnivore_chapparvovirus-MT123287.1-Chi
22	30.4	30.4	30.3	30.4	30.6	30.3	31.7	30.7	30.6	30.4	30.4	30.4	30.3	30.6	30.7	0.8	1.3	1.5	1.1	1.4	1.4		99.9	99.0	98.7	99.3	99.4	94.6	98.9	94.8	22	Carnivore_chapparvovirus-MT710947.1-Ita
23	30.4	30.4	30.4	30.4	30.7	30.4	31.8	30.8	30.7	30.4	30.5	30.4	30.4	30.7	30.7	0.9	1.4	1.6	1.2	1.5	1.5	0.1		98.9	98.6	99.2	99.3	94.6	98.8	94.8	23	Carnivore_chapparvovirus-MT710948.1-Ita
24	30.7	30.7	30.6	30.7	30.9	30.6	32.1	30.9	30.9	30.7	30.7	30.7	30.6	30.8	30.8	1.2	0.8	1.1	1.2	1.0	1.0	1.0	1.1		99.3	99.2	99.4	94.4	99.0	94.6	24	Carnivore_chapparvovirus-MN928790.1-Chi
25	30.8	31.0	30.9	31.0	31.2	30.7	32.4	31.1	31.2	30.8	30.9	30.8	30.7	31.2	31.3	1.2	1.0	1.3	1.3	0.9	0.9	1.3	1.4	0.7		98.9	99.1	94.2	98.7	94.4	25	Carnivore_chapparvovirus-MN928791.1-Chi
26	30.6	30.6	30.4	30.6	30.8	30.5	32.0	30.8	30.8	30.6	30.7	30.6	30.5	30.8	30.9	1.0	1.1	1.3	1.1	1.2	1.2	0.7	0.8	0.8	1.1		99.6	94.6	99.0	94.7	26	Carnivore_chapparvovirus-OK546100.1-Can
27	30.4	30.4	30.3	30.4	30.6	30.3	31.7	30.6	30.6	30.4	30.4	30.4	30.3	30.6	30.7	0.8	0.9	1.1	0.9	1.0	1.0	0.6	0.7	0.6	0.9	0.4		94.6	99.3	94.8	27	Carnivore_chapparvovirus-OK546101.1-Can
28	31.1	31.4	31.2	31.6	31.4	30.9	32.6	31.4	31.6	31.3	31.4	31.3	31.2	31.5	31.7	5.8	6.2	6.3	6.0	5.9	6.3	5.6	5.6	5.8	6.1	5.6	5.7		94.6	97.8	28	Carnivore_chapparvovirus-OK546102.1-Can
29	30.7	30.7	30.6	30.7	30.9	30.6	32.0	30.9	30.9	30.7	30.7	30.7	30.6	30.9	31.0	1.2	1.3	1.6	1.3	1.4	1.5	1.1	1.2	1.0	1.3	1.0	0.7	5.6		94.8	29	Carnivore_chapparvovirus-OM640108.1-Can
30	30.5	30.7	30.7	31.0	30.7	30.4	32.0	30.7	31.0	30.5	30.6	30.5	30.4	30.9	30.9	5.4	6.0	6.1	5.5	5.8	6.0	5.4	5.4	5.6	5.9	5.5	5.4	2.2	5.4		30	Carnivore_chapparvovirus-OM640109.1-Can
	1	2	3	4	5	6	7	8	9	10	11	12	13	14	15	16	17	18	19	20	21	22	23	24	25	26	27	28	29	30		

NS amino acid

Percent Identity

Divergence

	1	2	3	4	5	6	7	8	9	10	11	12	13	14	15	16	17	18	19	20	21	22	23	24	25	26	27	28	29	30		
1		99.2	98.9	99.4	97.9	97.9	97.0	98.5	99.5	97.9	97.6	97.7	97.9	98.8	97.7	66.3	66.0	65.7	66.1	65.7	65.7	66.6	66.4	66.4	65.8	66.6	66.7	66.6	66.6	67.0	1	Feline-chaphamaparvovirus-MN396757.1-Ca
2	0.8		98.8	99.2	97.7	97.7	97.1	98.3	99.7	97.7	97.4	97.6	97.7	98.6	97.6	65.9	65.6	65.3	65.7	65.3	65.3	66.2	66.0	66.0	65.4	66.2	66.3	66.2	66.2	66.6	2	Feline-chaphamaparvovirus-MN794869.1-US
3	1.1	1.2		98.9	97.7	97.4	96.5	98.0	99.1	97.4	97.1	97.3	97.4	98.3	97.3	66.3	66.0	65.7	66.1	65.7	65.7	66.6	66.4	66.4	65.8	66.6	66.7	66.6	66.6	67.0	3	Feline_chaphamaparvovirus-MW404251.1-It
4	0.6	0.8	1.1		97.9	97.9	97.0	98.5	99.5	97.9	97.6	97.7	97.9	98.8	97.7	66.1	65.8	65.5	66.0	65.5	65.5	66.4	66.3	66.3	65.7	66.4	66.6	66.4	66.4	66.9	4	Feline_chaphamaparvovirus-MW404252.1-It
5	2.2	2.3	2.3	2.2		97.6	95.4	98.5	98.0	97.6	97.3	97.4	97.6	97.3	96.8	65.7	65.2	64.9	65.3	64.9	64.9	65.8	65.7	65.7	65.0	65.8	66.0	65.8	65.8	66.3	5	Feline_chaphamaparvovirus-MW404253.1-It
6	2.2	2.3	2.6	2.2	2.5		95.4	99.1	98.0	99.7	99.7	99.8	99.7	97.9	98.0	66.1	65.7	65.3	65.8	65.3	65.3	66.3	66.1	66.1	65.8	66.3	66.4	66.3	66.3	66.7	6	Feline_chaphamaparvovirus-MT708230.1-Ch
7	3.1	2.9	3.6	3.1	4.7	4.7		96.0	97.4	95.4	95.1	95.3	95.4	96.4	95.3	64.4	64.1	63.8	64.4	64.0	63.8	64.7	64.6	64.6	64.0	64.7	64.9	64.6	64.7	65.0	7	Feline_chaphamaparvovirus-MT708231.1-Ch
8	1.5	1.7	2.0	1.5	1.5	0.9	4.1		98.6	98.8	98.8	98.9	98.8	97.9	97.7	65.8	65.3	65.0	65.5	65.0	65.0	66.0	65.8	65.8	65.5	66.0	66.1	66.0	66.0	66.4	8	Feline_chaphamaparvovirus-MZ031965.1-Ch
9	0.5	0.3	0.9	0.5	2.0	2.0	2.6	1.4		98.0	97.7	97.9	98.0	98.9	97.9	66.1	65.8	65.5	66.0	65.5	65.5	66.4	66.3	66.3	65.7	66.4	66.6	66.4	66.4	66.9	9	Feline_chaphamaparvovirus-MZ031966.1-Ch
10	2.2	2.3	2.6	2.2	2.5	0.3	4.7	1.2	2.0		99.7	99.8	100.0	97.9	98.0	66.3	65.8	65.5	66.0	65.5	65.5	66.4	66.3	66.3	66.0	66.4	66.6	66.4	66.4	66.9	10	Feline_chaphamaparvovirus-OP499830.1-Ch
11	2.5	2.6	2.9	2.5	2.8	0.3	5.0	1.2	2.3	0.3		99.8	99.7	97.6	97.7	66.1	65.7	65.3	65.8	65.3	65.3	66.3	66.1	66.1	65.8	66.3	66.4	66.3	66.3	66.7	11	Feline_chaphamaparvovirus-OP499831.1-Ch
12	2.3	2.5	2.8	2.3	2.6	0.2	4.9	1.1	2.2	0.2	0.2		99.8	97.7	97.9	66.1	65.7	65.3	65.8	65.3	65.3	66.3	66.1	66.1	65.8	66.3	66.4	66.3	66.3	66.7	12	Feline_chaphamaparvovirus-OP499832.1-Ch
13	2.2	2.3	2.6	2.2	2.5	0.3	4.7	1.2	2.0	0.0	0.3	0.2		97.9	98.0	66.3	65.8	65.5	66.0	65.5	65.5	66.4	66.3	66.3	66.0	66.4	66.6	66.4	66.4	66.9	13	Feline_chaphamaparvovirus-OP499833.1-Ch
14	1.2	1.4	1.7	1.2	2.8	2.2	3.7	2.2	1.1	2.2	2.5	2.3	2.2		98.9	65.8	65.5	65.2	65.7	65.2	65.2	66.1	66.0	66.0	65.3	66.1	66.3	66.1	66.1	66.6	14	Feline_chaphamaparvovirus-OQ162043.1-20
15	2.3	2.5	2.8	2.3	3.3	2.0	4.9	2.3	2.2	2.0	2.3	2.2	2.0	1.1		65.8	65.3	65.0	65.5	65.0	65.0	66.0	65.8	65.8	65.2	66.0	66.1	66.0	66.0	66.4	15	Feline_chaphamaparvovirus-OQ162042.1-20
16	44.7	45.4	44.7	44.9	45.7	44.9	48.0	45.5	44.9	44.7	44.9	44.9	44.7	45.5	45.5		98.2	97.7	98.6	98.5	98.3	98.9	98.8	98.9	98.6	99.1	99.2	96.4	98.6	96.4	16	Carnivore_chapparvovirus-MK448316.1-USA
17	45.2	45.9	45.2	45.5	46.6	45.7	48.5	46.3	45.5	45.5	45.7	45.7	45.5	46.0	46.3	1.8		98.3	97.9	98.0	97.9	98.6	98.5	98.9	98.2	98.8	98.9	96.4	98.3	96.1	17	Carnivore_chapparvovirus-MT123283.1-Chi
18	45.7	46.5	45.7	46.0	47.1	46.3	49.1	46.8	46.0	46.0	46.3	46.3	46.0	46.6	46.8	2.3	1.7		97.4	97.6	97.7	98.2	98.0	98.5	97.7	98.3	98.5	96.2	98.0	95.9	18	Carnivore_chapparvovirus-MT123284.1-Chi
19	44.9	45.6	44.9	45.2	46.3	45.5	48.0	46.0	45.2	45.2	45.5	45.5	45.2	45.7	46.0	1.4	2.1	2.6		98.9	98.2	98.6	98.5	98.6	98.5	98.8	98.9	96.4	98.3	96.1	19	Carnivore_chapparvovirus-MT123285.1-Chi
20	45.7	46.5	45.7	46.0	47.1	46.3	48.8	46.8	46.0	46.0	46.3	46.3	46.0	46.6	46.8	1.5	2.0	2.5	1.1		98.6	98.5	98.3	98.8	98.6	98.6	98.8	96.5	98.5	96.2	20	Carnivore_chapparvovirus-MT123286.1-Chi
21	45.7	46.5	45.7	46.0	47.1	46.3	49.1	46.8	46.0	46.0	46.3	46.3	46.0	46.6	46.8	1.7	2.1	2.3	1.8	1.4		98.3	98.2	98.6	98.5	98.5	98.6	96.2	98.0	95.9	21	Carnivore_chapparvovirus-MT123287.1-Chi
22	44.1	44.8	44.1	44.4	45.5	44.7	47.4	45.2	44.4	44.4	44.7	44.7	44.4	44.9	45.2	1.1	1.4	1.8	1.4	1.5	1.7		99.8	99.4	98.6	99.5	99.7	96.8	99.1	96.5	22	Carnivore_chapparvovirus-MT710947.1-Ita
23	44.4	45.1	44.4	44.7	45.7	44.9	47.7	45.5	44.7	44.7	44.9	44.9	44.7	45.2	45.5	1.2	1.5	2.0	1.5	1.7	1.8	0.2		99.2	98.5	99.4	99.5	96.7	98.9	96.4	23	Carnivore_chapparvovirus-MT710948.1-Ita
24	44.4	45.1	44.4	44.7	45.7	44.9	47.7	45.5	44.7	44.7	44.9	44.9	44.7	45.2	45.5	1.1	1.1	1.5	1.4	1.2	1.4	0.6	0.8		98.9	99.5	99.7	97.1	99.1	96.8	24	Carnivore_chapparvovirus-MN928790.1-Chi
25	45.5	46.2	45.5	45.7	46.8	45.5	48.8	46.0	45.7	45.2	45.5	45.5	45.2	46.3	46.6	1.4	1.8	2.3	1.5	1.4	1.5	1.4	1.5	1.1		98.8	98.9	96.4	98.3	96.1	25	Carnivore_chapparvovirus-MN928791.1-Chi
26	44.1	44.8	44.1	44.4	45.5	44.7	47.4	45.2	44.4	44.4	44.7	44.7	44.4	44.9	45.2	0.9	1.2	1.7	1.2	1.4	1.5	0.5	0.6	0.5	1.2		99.8	97.0	99.2	96.7	26	Carnivore_chapparvovirus-OK546100.1-Can
27	43.8	44.6	43.8	44.1	45.2	44.4	47.1	44.9	44.1	44.1	44.4	44.4	44.1	44.7	44.9	0.8	1.1	1.5	1.1	1.2	1.4	0.3	0.5	0.3	1.1	0.2		97.1	99.4	96.8	27	Carnivore_chapparvovirus-OK546101.1-Can
28	44.1	44.8	44.1	44.4	45.5	44.7	47.7	45.2	44.4	44.4	44.7	44.7	44.4	44.9	45.2	3.7	3.7	3.9	3.7	3.6	3.9	3.2	3.4	2.9	3.7	3.1	2.9		97.1	98.2	28	Carnivore_chapparvovirus-OK546102.1-Can
29	44.1	44.8	44.1	44.4	45.5	44.7	47.4	45.2	44.4	44.4	44.7	44.7	44.4	44.9	45.2	1.4	1.7	2.0	1.7	1.5	2.0	0.9	1.1	0.9	1.7	0.8	0.6	2.9		96.8	29	Carnivore_chapparvovirus-OM640108.1-Can
30	43.3	44.0	43.3	43.6	44.7	43.8	46.8	44.4	43.6	43.6	43.8	43.8	43.6	44.1	44.4	3.7	4.0	4.2	4.0	3.9	4.2	3.6	3.7	3.2	4.0	3.4	3.2	1.8	3.2		30	Carnivore_chapparvovirus-OM640109.1-Can
	1	2	3	4	5	6	7	8	9	10	11	12	13	14	15	16	17	18	19	20	21	22	23	24	25	26	27	28	29	30		

Percent Identity

Divergence

	1	2	3	4	5	6	7	8	9	10	11	12	13	14	15	16	17	18	19	20	21	22	23	24	25	26	27	28	29	30	31	32	33		
1		99.3	99.5	99.5	99.2	98.6	99.5	98.9	99.5	98.8	98.7	98.6	98.7	99.6	99.5	98.5	99.3	98.3	73.0	72.8	73.0	72.8	72.9	73.0	73.1	73.1	72.8	72.9	73.2	73.1	72.9	72.9	72.7	1	Feline_chaphamaparvovirus-MN396757.1-Ca
2	0.7		99.4	99.2	99.1	98.4	99.5	98.6	99.5	98.4	98.3	98.2	98.3	99.5	99.4	98.1	98.8	98.0	72.9	72.7	72.9	72.7	72.9	72.9	73.0	73.0	72.9	72.9	73.1	73.1	72.8	72.8	72.6	2	Feline_chaphamaparvovirus-MN794869.1-US
3	0.5	0.6		99.5	99.5	98.4	99.6	98.7	99.6	98.6	98.5	98.4	98.5	99.8	99.7	98.4	99.0	98.1	73.0	72.9	73.1	72.9	73.0	73.1	73.1	73.1	72.9	73.0	73.3	73.2	72.7	72.9	72.6	3	Feline_chaphamaparvovirus-MW404251.1-It
4	0.5	0.8	0.5		99.2	98.4	99.4	98.9	99.4	98.5	98.4	98.3	98.4	99.6	99.5	98.4	99.0	98.2	73.1	72.9	73.1	72.9	73.1	73.3	73.2	73.2	73.1	73.1	73.3	73.3	73.0	73.0	72.7	4	Feline_chaphamaparvovirus-MW404252.1-It
5	0.8	0.9	0.5	0.8		98.2	99.3	98.5	99.3	98.2	98.3	98.2	98.3	99.5	99.4	98.1	98.6	97.8	73.0	72.9	73.1	72.9	73.0	73.1	73.1	73.1	72.9	73.1	73.3	73.2	72.7	72.9	72.6	5	Feline_chaphamaparvovirus-MW404253.1-It
6	1.4	1.6	1.6	1.7	1.8		98.8	98.8	98.8	98.7	98.8	98.6	98.8	98.5	98.4	98.0	98.4	98.1	72.8	72.4	72.6	72.4	72.6	72.6	72.7	72.7	72.4	72.6	72.9	72.8	72.4	72.5	72.2	6	Feline_chaphamaparvovirus-MT708230.1-Ch
7	0.5	0.5	0.4	0.6	0.7	1.2		98.8	100.0	98.6	98.5	98.4	98.5	99.7	99.6	98.3	99.0	98.2	73.2	73.0	73.2	73.0	73.1	73.2	73.3	73.3	73.0	73.1	73.4	73.3	72.9	73.1	72.8	7	Feline_chaphamaparvovirus-MT708231.1-Ch
8	1.1	1.5	1.3	1.1	1.5	1.2	1.2		98.8	99.1	99.1	99.0	99.1	98.8	98.7	98.6	99.1	98.5	72.8	72.6	72.8	72.7	72.7	72.8	72.9	72.9	72.6	72.7	73.0	72.9	72.5	72.6	72.4	8	Feline_chaphamaparvovirus-MZ031965.1-Ch
9	0.5	0.5	0.4	0.6	0.7	1.2	0.0	1.2		98.6	98.5	98.4	98.5	99.7	99.6	98.3	99.0	98.2	73.2	73.0	73.2	73.0	73.1	73.2	73.3	73.3	73.0	73.1	73.4	73.3	72.9	73.1	72.8	9	Feline_chaphamaparvovirus-MZ031966.1-Ch
10	1.3	1.7	1.5	1.5	1.8	1.3	1.5	0.9	1.5		99.9	99.8	99.9	98.6	98.6	98.7	99.0	98.5	72.5	72.3	72.5	72.3	72.4	72.5	72.6	72.6	72.3	72.4	72.7	72.6	72.2	72.4	72.1	10	Feline_chaphamaparvovirus-OP499830.1-Ch
11	1.3	1.7	1.5	1.6	1.7	1.3	1.5	0.9	1.5	0.1		99.9	100.0	98.6	98.5	98.6	98.9	98.4	72.6	72.3	72.5	72.3	72.4	72.5	72.6	72.6	72.3	72.4	72.7	72.6	72.2	72.4	72.1	11	Feline_chaphamaparvovirus-OP499831.1-Ch
12	1.5	1.9	1.7	1.7	1.9	1.4	1.7	1.0	1.7	0.2	0.1		99.9	98.4	98.4	98.5	98.8	98.3	72.5	72.2	72.4	72.2	72.4	72.4	72.5	72.5	72.2	72.4	72.6	72.6	72.2	72.3	72.0	12	Feline_chaphamaparvovirus-OP499832.1-Ch
13	1.3	1.7	1.5	1.6	1.7	1.3	1.5	0.9	1.5	0.1	0.0	0.1		98.6	98.5	98.6	98.9	98.4	72.6	72.3	72.5	72.3	72.4	72.5	72.6	72.6	72.3	72.4	72.7	72.6	72.2	72.4	72.1	13	Feline_chaphamaparvovirus-OP499833.1-Ch
14	0.4	0.5	0.2	0.4	0.5	1.5	0.3	1.3	0.3	1.4	1.5	1.6	1.5		99.8	98.5	99.0	98.2	73.1	72.9	73.1	72.9	73.1	73.1	73.2	73.2	72.9	73.1	73.3	73.3	72.9	73.0	72.7	14	Feline_chaphamaparvovirus-MW404254.1-It
15	0.5	0.6	0.3	0.5	0.6	1.6	0.4	1.3	0.4	1.5	1.5	1.7	1.5	0.2		98.4	99.0	98.1	73.0	72.8	73.0	72.8	72.9	73.0	73.1	73.1	72.8	72.9	73.2	73.1	72.7	72.9	72.6	15	Feline_chaphamaparvovirus-MW404255.1-It
16	1.5	1.9	1.6	1.7	1.9	2.0	1.7	1.5	1.7	1.3	1.4	1.5	1.4	1.5	1.6		98.3	97.8	72.5	72.3	72.5	72.3	72.4	72.5	72.6	72.6	72.3	72.4	72.7	72.6	72.2	72.4	72.1	16	Feline_chaphamaparvovirus-MZ712100.1-Tu
17	0.7	1.3	1.1	1.0	1.4	1.6	1.1	0.9	1.1	1.1	1.1	1.3	1.1	1.0	1.1	1.7		99.0	72.8	72.6	72.8	72.6	72.7	72.8	72.9	72.9	72.6	72.7	73.0	72.9	72.6	72.6	72.6	17	Feline_chaphamaparvovirus-OQ162043.1-20
18	1.7	2.0	1.9	1.9	2.3	1.9	1.8	1.5	1.8	1.5	1.6	1.7	1.6	1.9	1.9	2.2	1.0		72.7	72.5	72.6	72.5	72.6	72.7	72.8	72.8	72.6	72.6	72.9	72.9	72.3	72.5	72.2	18	Feline_chaphamaparvovirus-OQ162042.1-20
19	33.6	33.7	33.5	33.4	33.5	33.9	33.3	33.8	33.3	34.3	34.2	34.3	34.2	33.4	33.6	34.3	33.9	34.0		98.7	98.9	99.0	98.6	99.1	99.1	99.1	98.7	98.9	99.1	99.5	92.9	99.0	92.6	19	Carnivore_chapparvovirus-MK448316.1-USA
20	33.8	33.9	33.7	33.6	33.7	34.4	33.5	34.2	33.5	34.6	34.6	34.7	34.6	33.6	33.8	34.6	34.2	34.3	1.3		98.2	98.2	98.3	98.4	99.2	99.3	98.0	98.1	98.6	98.9	92.7	98.3	92.5	20	Carnivore_chapparvovirus-MT123283.1-Chi
21	33.5	33.6	33.4	33.3	33.4	34.1	33.2	33.8	33.2	34.3	34.3	34.4	34.3	33.3	33.5	34.3	33.8	34.1	1.1	1.9		98.9	98.6	99.2	98.5	98.6	98.9	99.4	98.7	99.1	92.6	98.9	92.3	21	Carnivore_chapparvovirus-MT123284.1-Chi
22	33.8	33.9	33.7	33.6	33.7	34.4	33.5	33.9	33.5	34.6	34.6	34.7	34.6	33.6	33.8	34.6	34.2	34.3	1.0	1.8	1.1		98.8	99.1	98.6	98.7	98.7	98.8	98.8	99.2	92.3	98.8	92.1	22	Carnivore_chapparvovirus-MT123285.1-Chi
23	33.6	33.7	33.5	33.4	33.5	34.2	33.3	33.9	33.3	34.4	34.4	34.5	34.4	33.4	33.6	34.4	33.9	34.1	1.4	1.7	1.4	1.2		98.9	98.7	98.8	98.5	98.5	98.5	98.9	92.4	98.5	92.1	23	Carnivore_chapparvovirus-MT123286.1-Chi
24	33.5	33.6	33.4	33.1	33.4	34.1	33.2	33.8	33.2	34.3	34.3	34.4	34.3	33.3	33.5	34.3	33.8	34.0	0.9	1.6	0.8	0.9	1.1		98.8	98.9	99.2	99.1	99.0	99.4	92.8	99.0	92.4	24	Carnivore_chapparvovirus-MT123287.1-Chi
25	33.4	33.5	33.3	33.2	33.3	34.0	33.1	33.7	33.1	34.2	34.2	34.3	34.2	33.2	33.4	34.2	33.7	33.8	0.9	0.8	1.5	1.4	1.3	1.2		99.9	98.4	98.5	98.9	99.1	93.1	98.7	92.9	25	Carnivore_chapparvovirus-MT710947.1-Ita
26	33.4	33.5	33.3	33.2	33.3	34.0	33.1	33.7	33.1	34.2	34.2	34.3	34.2	33.2	33.4	34.2	33.7	33.8	0.9	0.7	1.4	1.3	1.2	1.1	0.1		98.5	98.5	98.9	99.2	93.2	98.8	92.9	26	Carnivore_chapparvovirus-MT710948.1-Ita
27	33.8	33.7	33.7	33.4	33.7	34.4	33.5	34.2	33.5	34.6	34.6	34.7	34.6	33.6	33.8	34.6	34.2	34.1	1.3	2.0	1.1	1.3	1.5	0.8	1.6	1.5		98.9	98.6	99.0	92.5	98.6	92.1	27	Carnivore_chapparvovirus-MN928790.1-Chi
28	33.6	33.7	33.5	33.4	33.3	34.2	33.3	33.9	33.3	34.4	34.4	34.5	34.4	33.4	33.6	34.4	33.9	34.2	1.1	1.9	0.6	1.2	1.5	0.9	1.5	1.5	1.1		98.7	99.1	92.5	98.8	92.3	28	Carnivore_chapparvovirus-MN928791.1-Chi
29	33.2	33.3	33.1	33.0	33.1	33.7	32.9	33.5	32.9	34.0	34.0	34.1	34.0	33.0	33.2	33.9	33.5	33.6	0.9	1.4	1.3	1.2	1.5	1.0	1.1	1.1	1.4	1.3		99.6	92.8	98.9	92.5	29	Carnivore_chapparvovirus-OK546100.1-Can
30	33.3	33.4	33.2	33.1	33.2	33.8	33.0	33.6	33.0	34.1	34.1	34.2	34.1	33.1	33.3	34.1	33.6	33.7	0.5	1.1	0.9	0.8	1.1	0.6	0.9	0.8	1.0	0.9	0.4		92.9	99.3	92.7	30	Carnivore_chapparvovirus-OK546101.1-Can
31	33.7	33.8	34.0	33.5	33.9	34.4	33.6	34.3	33.6	34.7	34.7	34.8	34.7	33.7	34.0	34.7	34.1	34.6	7.6	7.8	7.9	8.2	8.1	7.6	7.3	7.2	7.9	7.9	7.6	7.5		92.8	97.7	31	Carnivore_chapparvovirus-OK546102.1-Can
32	33.7	33.8	33.6	33.5	33.6	34.3	33.4	34.1	33.4	34.5	34.5	34.6	34.5	33.5	33.7	34.5	34.1	34.3	1.0	1.7	1.1	1.2	1.5	1.0	1.3	1.2	1.4	1.2	1.1	0.7	7.6		92.5	32	Carnivore_chapparvovirus-OM640108.1-Can
33	34.0	34.1	34.2	34.0	34.2	34.7	33.8	34.5	33.8	34.9	34.9	35.0	34.9	34.0	34.2	34.9	34.2	34.8	7.9	7.9	8.2	8.5	8.4	8.1	7.6	7.5	8.4	8.2	7.9	7.8	2.4	7.9		33	Carnivore_chapparvovirus-OM640109.1-Can
	1	2	3	4	5	6	7	8	9	10	11	12	13	14	15	16	17	18	19	20	21	22	23	24	25	26	27	28	29	30	31	32	33		

VP amino acid

Percent Identity

Divergence

	1	2	3	4	5	6	7	8	9	10	11	12	13	14	15	16	17	18	19	20	21	22	23	24	25	26	27	28	29	30	31	32	33		
1		99.6	99.6	99.8	98.6	97.6	99.8	98.4	99.8	98.6	98.4	98.2	98.4	99.6	99.6	97.0	98.8	97.2	68.9	68.6	69.1	69.1	69.5	69.3	69.5	69.5	68.9	68.9	69.5	69.5	69.1	69.1	68.9	1	Feline_chaphamaparvovirus-MN396757.1-Ca
2	0.4		99.2	99.4	98.2	97.4	99.4	98.2	99.4	98.2	98.0	97.8	98.0	99.2	99.2	96.9	98.4	96.9	68.7	68.4	68.9	68.9	69.3	69.1	69.3	69.3	68.6	68.6	69.3	69.3	68.9	68.9	68.6	2	Feline_chaphamaparvovirus-MN794869.1-US
3	0.4	0.8		99.8	98.6	97.2	99.4	98.0	99.4	98.2	98.0	97.8	98.0	99.6	99.6	97.0	98.4	96.9	68.5	68.2	68.6	68.6	69.1	68.9	69.1	69.1	68.4	68.4	69.1	69.1	68.6	68.6	68.4	3	Feline_chaphamaparvovirus-MW404251.1-It
4	0.2	0.6	0.2		98.8	97.4	99.6	98.2	99.6	98.4	98.2	98.0	98.2	99.8	99.8	97.2	98.6	97.0	68.7	68.4	68.9	68.9	69.3	69.1	69.3	69.3	68.6	68.6	69.3	69.3	68.9	68.9	68.6	4	Feline_chaphamaparvovirus-MW404252.1-It
5	1.4	1.8	1.4	1.2		96.7	98.4	97.4	98.4	97.2	97.4	97.2	97.4	98.6	98.6	96.1	97.4	95.9	68.5	68.2	68.6	68.6	69.1	68.9	69.1	69.1	68.4	68.4	69.1	69.1	68.6	68.6	68.4	5	Feline_chaphamaparvovirus-MW404253.1-It
6	2.4	2.6	2.8	2.6	3.4		97.8	98.4	97.8	97.8	98.0	97.8	98.0	97.2	97.2	96.3	97.2	96.5	68.7	68.2	68.6	68.6	69.1	68.9	69.1	69.1	68.2	68.4	69.1	69.1	68.6	68.6	68.4	6	Feline_chaphamaparvovirus-MT708230.1-Ch
7	0.2	0.6	0.6	0.4	1.6	2.2		98.2	100.0	98.4	98.2	98.0	98.2	99.4	99.4	96.9	98.6	97.0	69.1	68.9	69.3	69.3	69.7	69.5	69.7	69.7	69.1	69.1	69.7	69.7	69.3	69.3	69.1	7	Feline_chaphamaparvovirus-MT708231.1-Ch
8	1.6	1.8	2.0	1.8	2.6	1.6	1.8		98.2	99.4	99.6	99.4	99.6	98.0	98.0	97.4	98.0	97.2	68.5	68.0	68.4	68.4	68.9	68.6	68.9	68.9	68.2	68.2	68.9	68.9	68.4	68.4	68.2	8	Feline_chaphamaparvovirus-MZ031965.1-Ch
9	0.2	0.6	0.6	0.4	1.6	2.2	0.0	1.8		98.4	98.2	98.0	98.2	99.4	99.4	96.9	98.6	97.0	69.1	68.9	69.3	69.3	69.7	69.5	69.7	69.7	69.1	69.1	69.7	69.7	69.3	69.3	69.1	9	Feline_chaphamaparvovirus-MZ031966.1-Ch
10	1.4	1.8	1.8	1.6	2.8	2.2	1.6	0.6	1.6		99.8	99.6	99.8	98.2	98.2	98.0	98.2	97.4	68.7	68.4	68.9	68.9	69.3	69.1	69.3	69.3	68.6	68.6	69.3	69.3	68.9	68.9	68.6	10	Feline_chaphamaparvovirus-OP499830.1-Ch
11	1.6	2.0	2.0	1.8	2.6	2.0	1.8	0.4	1.8	0.2		99.8	100.0	98.0	98.0	97.8	98.0	97.2	68.9	68.4	68.9	68.9	69.3	69.1	69.3	69.3	68.6	68.6	69.3	69.3	68.9	68.9	68.6	11	Feline_chaphamaparvovirus-OP499831.1-Ch
12	1.8	2.2	2.2	2.0	2.8	2.2	2.0	0.6	2.0	0.4	0.2		99.8	97.8	97.8	97.6	97.8	97.0	68.9	68.4	68.9	68.9	69.3	69.1	69.3	69.3	68.6	68.6	69.3	69.3	68.9	68.9	68.6	12	Feline_chaphamaparvovirus-OP499832.1-Ch
13	1.6	2.0	2.0	1.8	2.6	2.0	1.8	0.4	1.8	0.2	0.0	0.2		98.0	98.0	97.8	98.0	97.2	68.9	68.4	68.9	68.9	69.3	69.1	69.3	69.3	68.6	68.6	69.3	69.3	68.9	68.9	68.6	13	Feline_chaphamaparvovirus-OP499833.1-Ch
14	0.4	0.8	0.4	0.2	1.4	2.8	0.6	2.0	0.6	1.8	2.0	2.2	2.0		99.6	97.0	98.4	96.9	68.7	68.4	68.9	68.9	69.3	69.1	69.3	69.3	68.6	68.6	69.3	69.3	68.9	68.9	68.6	14	Feline_chaphamaparvovirus-MW404254.1-It
15	0.4	0.8	0.4	0.2	1.4	2.8	0.6	2.0	0.6	1.8	2.0	2.2	2.0	0.4		97.0	98.4	96.9	68.7	68.4	68.9	68.9	69.3	69.1	69.3	69.3	68.6	68.6	69.3	69.3	68.9	68.9	68.6	15	Feline_chaphamaparvovirus-MW404255.1-It
16	3.0	3.2	3.0	2.8	4.0	3.8	3.2	2.6	3.2	2.0	2.2	2.4	2.2	3.0	3.0		96.3	95.5	67.9	67.6	68.0	68.0	68.4	68.2	68.4	68.4	67.8	67.8	68.4	68.4	68.0	68.0	67.8	16	Feline_chaphamaparvovirus-MZ712100.1-Tu
17	1.2	1.6	1.6	1.4	2.6	2.8	1.4	2.0	1.4	1.8	2.0	2.2	2.0	1.6	1.6	3.8		98.4	68.3	68.0	68.4	68.4	68.9	68.6	68.9	68.9	68.2	68.2	68.9	68.9	68.4	68.4	68.2	17	Feline_chaphamaparvovirus-OQ162043.1-20
18	2.8	3.2	3.2	3.0	4.3	3.6	3.0	2.8	3.0	2.6	2.8	3.0	2.8	3.2	3.2	4.7	1.6		68.3	68.0	68.4	68.4	68.9	68.6	68.9	68.9	68.6	68.2	68.9	68.9	68.4	68.4	68.2	18	Feline_chaphamaparvovirus-OQ162042.1-20
19	40.2	40.5	40.8	40.5	40.8	40.5	39.8	40.8	39.8	40.5	40.2	40.2	40.2	40.5	40.5	41.9	41.2	41.2		98.8	99.2	99.0	99.2	99.4	99.4	99.6	98.8	98.8	99.6	99.8	98.6	99.0	98.4	19	Carnivore_chapparvovirus-MK448316.1-USA
20	40.5	40.9	41.2	40.9	41.2	41.2	40.2	41.6	40.2	40.9	40.9	40.9	40.9	40.9	40.9	42.3	41.6	41.6	1.2		98.4	98.6	98.8	98.6	99.0	99.2	98.0	98.0	98.8	99.0	97.8	98.2	97.6	20	Carnivore_chapparvovirus-MT123283.1-Chi
21	39.8	40.2	40.5	40.2	40.5	40.5	39.5	40.9	39.5	40.2	40.2	40.2	40.2	40.2	40.2	41.6	40.9	40.9	0.8	1.6		98.6	98.8	99.0	99.0	99.2	98.8	99.2	99.2	99.4	98.4	99.0	98.2	21	Carnivore_chapparvovirus-MT123284.1-Chi
22	39.8	40.2	40.5	40.2	40.5	40.5	39.5	40.9	39.5	40.2	40.2	40.2	40.2	40.2	40.2	41.6	40.9	40.9	1.0	1.4	1.4		99.4	98.8	99.2	99.4	98.2	98.2	99.0	99.2	98.0	98.4	97.8	22	Carnivore_chapparvovirus-MT123285.1-Chi
23	39.2	39.5	39.8	39.5	39.8	39.8	38.8	40.2	38.8	39.5	39.5	39.5	39.5	39.5	39.5	40.9	40.2	40.2	0.8	1.2	1.2	0.6		99.0	99.4	99.6	98.4	98.4	99.2	99.4	98.2	98.6	98.0	23	Carnivore_chapparvovirus-MT123286.1-Chi
24	39.5	39.8	40.2	39.8	40.2	40.2	39.2	40.5	39.2	39.8	39.8	39.8	39.8	39.8	39.8	41.2	40.5	40.5	0.6	1.4	1.0	1.2	1.0		99.2	99.4	98.6	98.6	99.4	99.6	98.4	98.8	98.2	24	Carnivore_chapparvovirus-MT123287.1-Chi
25	39.2	39.5	39.8	39.5	39.8	39.8	38.8	40.2	38.8	39.5	39.5	39.5	39.5	39.5	39.5	40.9	40.2	40.2	0.6	1.0	1.0	0.8	0.6	0.8		99.8	98.6	98.6	99.4	99.6	98.4	98.8	98.2	25	Carnivore_chapparvovirus-MT710947.1-Ita
26	39.2	39.5	39.8	39.5	39.8	39.8	38.8	40.2	38.8	39.5	39.5	39.5	39.5	39.5	39.5	40.9	40.2	40.2	0.4	0.8	0.8	0.6	0.4	0.6	0.2		98.8	98.8	99.6	99.8	98.6	99.0	98.4	26	Carnivore_chapparvovirus-MT710948.1-Ita
27	40.2	40.5	40.9	40.5	40.9	41.2	39.8	41.2	39.8	40.5	40.5	40.5	40.5	40.5	40.5	41.9	41.2	40.5	1.2	2.0	1.2	1.8	1.6	1.4	1.4	1.2		98.4	98.8	99.0	97.8	98.2	97.6	27	Carnivore_chapparvovirus-MN928790.1-Chi
28	40.2	40.5	40.9	40.5	40.9	40.9	39.8	41.2	39.8	40.5	40.5	40.5	40.5	40.5	40.5	41.9	41.2	41.2	1.2	2.0	0.8	1.8	1.6	1.4	1.4	1.2	1.6		98.8	99.0	98.2	98.6	97.8	28	Carnivore_chapparvovirus-MN928791.1-Chi
29	39.2	39.5	39.8	39.5	39.8	39.8	38.8	40.2	38.8	39.5	39.5	39.5	39.5	39.5	39.5	40.9	40.2	40.2	0.4	1.2	0.8	1.0	0.8	0.6	0.6	0.4	1.2	1.2		99.8	98.6	99.0	98.4	29	Carnivore_chapparvovirus-OK546100.1-Can
30	39.2	39.5	39.8	39.5	39.8	39.8	38.8	40.2	38.8	39.5	39.5	39.5	39.5	39.5	39.5	40.9	40.2	40.2	0.2	1.0	0.6	0.8	0.6	0.4	0.4	0.2	1.0	1.0	0.2		98.8	99.2	98.6	30	Carnivore_chapparvovirus-OK546101.1-Can
31	39.8	40.2	40.5	40.2	40.5	40.5	39.5	40.9	39.5	40.2	40.2	40.2	40.2	40.2	40.2	41.6	40.9	40.9	1.4	2.2	1.6	2.0	1.8	1.6	1.6	1.4	2.2	1.8	1.4	1.2		98.6	99.4	31	Carnivore_chapparvovirus-OK546102.1-Can
32	39.8	40.2	40.5	40.2	40.5	40.5	39.5	40.9	39.5	40.2	40.2	40.2	40.2	40.2	40.2	41.6	40.9	40.9	1.0	1.8	1.0	1.6	1.4	1.2	1.2	1.0	1.8	1.4	1.0	0.8	1.4		98.4	32	Carnivore_chapparvovirus-OM640108.1-Can
33	40.2	40.5	40.9	40.5	40.9	40.9	39.8	41.2	39.8	40.5	40.5	40.5	40.5	40.5	40.5	41.9	41.2	41.2	1.6	2.4	1.8	2.2	2.0	1.8	1.8	1.6	2.4	2.2	1.6	1.4	0.6	1.6		33	Carnivore_chapparvovirus-OM640109.1-Can
	1	2	3	4	5	6	7	8	9	10	11	12	13	14	15	16	17	18	19	20	21	22	23	24	25	26	27	28	29	30	31	32	33		
