# Supplementary material for: Phylogenetic Analysis and Codon Usage Bias Reveal the Base of Feline and Canine Chaphamaparvovirus for Cross-Species Transmission
Source: Animals (Basel). 2023 Aug 14;13(16):2617. doi: 10.3390/ani13162617 (PMC10451695; doi:10.3390/ani13162617)
Supplement: Supplementary file 1 [file animals-13-02617-s001.zip › Supplementary Table 5.pdf]

Supplementary Table 5

Detailed information of relative synonymous codon usage analysis (RSCU) of FeChPV and CaChPV with hosts.

Codons		Virus				Host	
		FeChPV		CaChPV		Canis	Felis catus
		NS	VP	NS	VP		
TTT	F	<u>1.527</u>	0.911	**<u>1.740</u>**	**<u>1.953</u>**	0.961	0.921
TTC	F	0.473	<u>1.089</u>	0.260	0.047	<u>1.039</u>	<u>1.079</u>
TTA	L	**<u>2.655</u>**	**<u>2.758</u>**	**<u>2.583</u>**	**<u>3.076</u>**	0.519	0.479
TTG	L	<u>1.556</u>	0.249	<u>1.075</u>	0.245	0.804	0.787
CTT	L	0.529	0.748	<u>1.558</u>	0.971	0.827	0.784
CTC	L	0.397	0.249	0.160	0.474	<u>1.131</u>	<u>1.172</u>
CTA	L	0.712	<u>1.247</u>	0.552	<u>1.217</u>	0.457	0.418
CTG	L	0.153	0.748	0.072	0.017	**<u>2.263</u>**	**<u>2.359</u>**
ATT	I	<u>1.597</u>	0.874	<u>1.565</u>	**<u>1.729</u>**	<u>1.100</u>	<u>1.055</u>
ATC	I	0.309	0.426	0.522	0.206	<u>1.349</u>	<u>1.422</u>
ATA	I	<u>1.094</u>	**<u>1.699</u>**	0.913	<u>1.065</u>	0.551	0.523
GTT	V	**<u>2.021</u>**	0.547	**<u>1.614</u>**	**<u>1.865</u>**	0.751	0.719
GTC	V	0.598	<u>1.096</u>	0.192	0.113	0.936	0.972
GTA	V	0.801	**<u>2.291</u>**	**<u>1.644</u>**	**<u>1.838</u>**	0.514	0.471
GTG	V	0.579	0.067	0.550	0.184	**<u>1.799</u>**	**<u>1.838</u>**
TCT	S	<u>1.479</u>	0.875	**<u>2.007</u>**	**<u>1.969</u>**	<u>1.146</u>	<u>1.088</u>

TCC	S	0.664	0.013	0.825	0.415	1.249	1.312
TCA	S	1.397	**2.676**	1.216	**1.862**	0.916	0.854
TCG	S	0.082	0.232	0.096	0.216	0.333	0.385
AGT	S	0.594	1.249	0.645	0.574	0.953	0.906
AGC	S	**1.783**	0.954	1.211	0.963	1.403	1.455
CCT	P	1.365	0.908	**2.224**	1.510	1.147	1.091
CCC	P	0.463	0.007	0.672	0.562	1.276	1.337
CCA	P	**1.971**	**2.991**	1.103	**1.727**	1.108	1.027
CCG	P	0.201	0.093	0.000	0.201	0.468	0.546
ACT	T	**1.758**	1.260	1.493	1.301	1.010	0.940
ACC	T	0.736	0.440	0.796	0.367	1.352	1.420
ACA	T	1.236	**2.107**	1.499	**1.958**	1.164	1.072
ACG	T	0.269	0.193	0.212	0.374	0.474	0.569
GCT	A	**2.286**	1.334	**1.608**	**1.547**	1.066	0.993
GCC	A	0.582	0.000	0.471	0.143	1.581	**1.655**
GCA	A	1.132	**2.515**	**1.626**	**2.276**	0.933	0.859
GCG	A	0.000	0.151	0.295	0.035	0.421	0.493
TAT	Y	**1.720**	1.451	**1.874**	1.281	0.920	0.865
TAC	Y	0.280	0.549	0.126	0.719	1.080	1.135
CAT	H	1.270	1.595	1.540	**1.679**	0.861	0.792
CAC	H	0.730	0.405	0.460	0.321	1.139	1.208
CAA	Q	1.483	**1.680**	1.562	**1.723**	0.542	0.510
CAG	Q	0.517	0.320	0.438	0.277	1.458	1.490
AAT	N	1.345	1.585	1.134	1.542	0.978	0.912
AAC	N	0.655	0.415	0.866	0.458	1.022	1.088
AAA	K	**1.600**	**1.986**	**1.917**	**1.871**	0.898	0.872

AAG	K	0.400	0.014	0.083	0.129	1.102	1.128
GAT	D	1.573	1.281	**1.637**	1.490	0.966	0.899
GAC	D	0.427	0.719	0.363	0.510	1.034	1.101
GAA	E	**1.749**	**1.895**	**1.639**	**1.994**	0.886	0.850
GAG	E	0.251	0.105	0.361	0.006	1.114	1.150
TGT	C	1.000	0.875	1.084	**1.721**	0.957	0.933
TGC	C	1.000	1.125	0.916	0.279	1.043	1.067
CGT	R	0.435	0.364	0.927	0.938	0.466	0.463
CGC	R	0.435	0.000	0.595	0.560	1.003	1.056
CGA	R	0.590	0.000	1.288	1.420	0.673	0.659
CGG	R	0.156	0.000	0.156	0.059	1.217	1.285
AGA	R	**3.948**	**5.553**	**2.255**	**2.605**	1.351	1.260
AGG	R	0.435	0.083	0.780	0.419	1.291	1.277
GGT	G	0.738	0.582	0.657	1.038	0.653	0.631
GGC	G	0.485	0.143	0.746	0.443	1.319	1.358
GGA	G	**2.008**	**2.980**	**2.117**	**2.485**	1.026	0.992
GGG	G	0.768	0.295	0.480	0.033	1.002	1.019

Note:RSCU > 1.6 are underlined and **bolde**d; 1<rscu<1.6 are underlined.
