# Supplementary material for: Phylogenetic Analysis and Codon Usage Bias Reveal the Base of Feline and Canine Chaphamaparvovirus for Cross-Species Transmission
Source: Animals (Basel). 2023 Aug 14;13(16):2617. doi: 10.3390/ani13162617 (PMC10451695; doi:10.3390/ani13162617)
Supplement: Supplementary file 1 [file animals-13-02617-s001.zip › Supplementary Table 6.pdf]

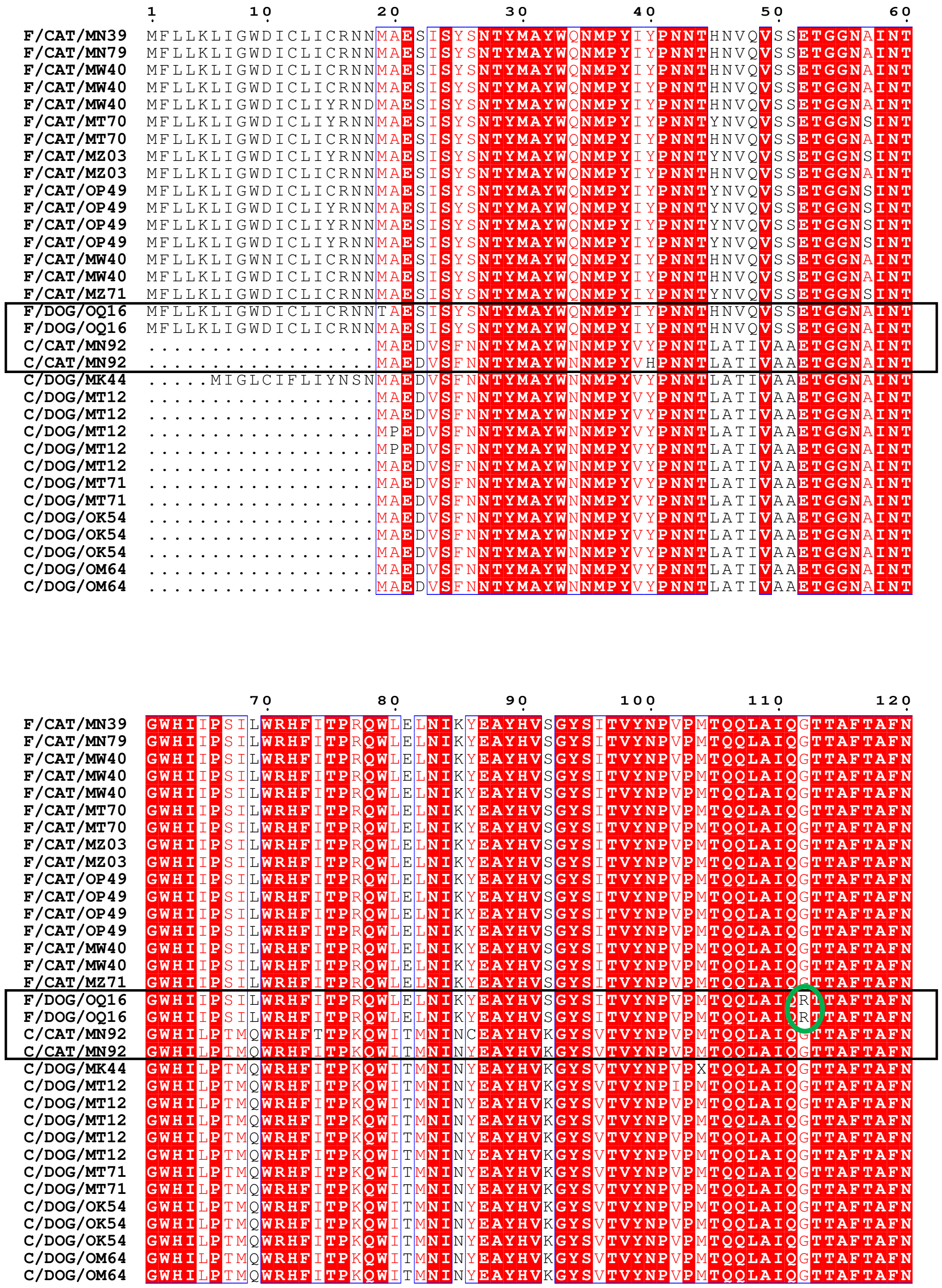
F/CAT/MN39
F/CAT/MN79
F/CAT/MW40
F/CAT/MW40
F/CAT/MW40
F/CAT/MT70
F/CAT/MT70
F/CAT/MZ03
F/CAT/MZ03
F/CAT/OP49
F/CAT/OP49
F/CAT/OP49
F/CAT/OP49
F/CAT/MW40
F/CAT/MW40
F/CAT/MZ71
F/DOG/OQ16
F/DOG/OQ16
C/CAT/MN92
C/CAT/MN92
C/DOG/MK44
C/DOG/MT12
C/DOG/MT12
C/DOG/MT12
C/DOG/MT12
C/DOG/MT12
C/DOG/MT71
C/DOG/MT71
C/DOG/OK54
C/DOG/OK54
C/DOG/OK54
C/DOG/OM64
C/DOG/OM64

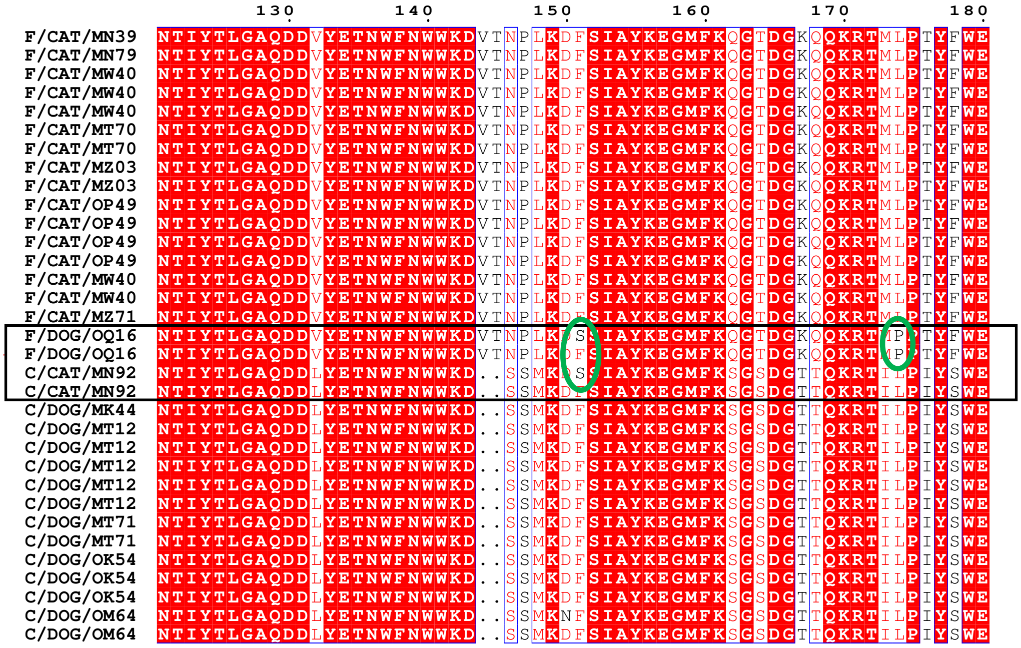
130
140
150
160
170
180
F/CAT/MN39
F/CAT/MN79
F/CAT/MW40
F/CAT/MW40
F/CAT/MW40
F/CAT/MT70
F/CAT/MT70
F/CAT/MZ03
F/CAT/MZ03
F/CAT/OP49
F/CAT/OP49
F/CAT/OP49
F/CAT/OP49
F/CAT/MW40
F/CAT/MW40
F/CAT/MZ71
F/DOG/OQ16
F/DOG/OQ16
C/CAT/MN92
C/CAT/MN92
C/DOG/MK44
C/DOG/MT12
C/DOG/MT12
C/DOG/MT12
C/DOG/MT12
C/DOG/MT12
C/DOG/MT71
C/DOG/MT71
C/DOG/OK54
C/DOG/OK54
C/DOG/OK54
C/DOG/OM64
C/DOG/OM64

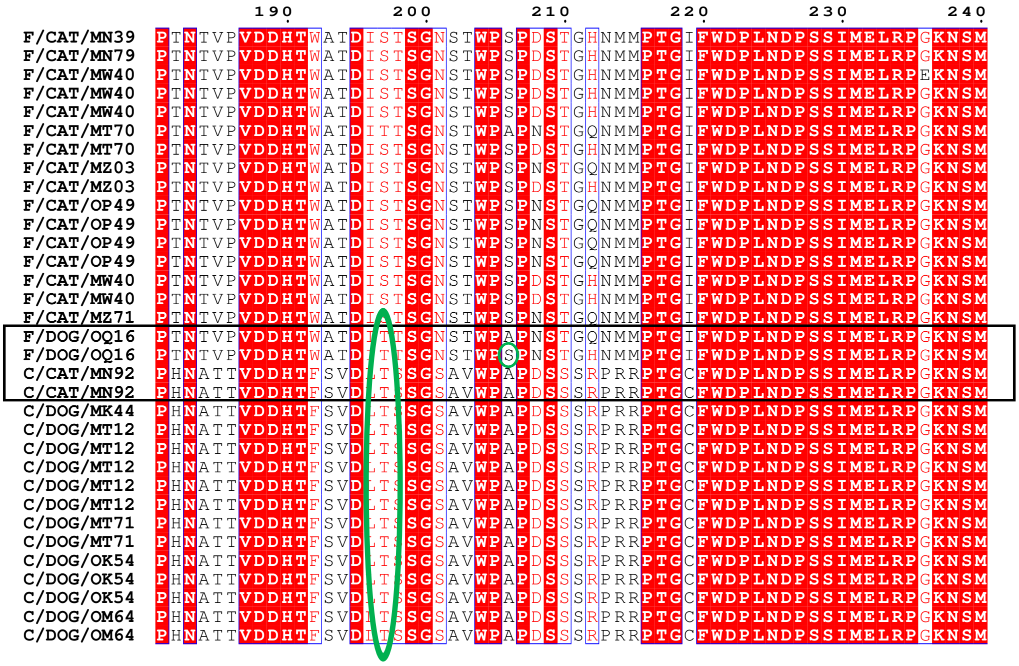
190
200
210
220
230
240
F/CAT/MN39
F/CAT/MN79
F/CAT/MW40
F/CAT/MW40
F/CAT/MW40
F/CAT/MT70
F/CAT/MT70
F/CAT/MZ03
F/CAT/MZ03
F/CAT/OP49
F/CAT/OP49
F/CAT/OP49
F/CAT/OP49
F/CAT/MW40
F/CAT/MW40
F/CAT/MZ71
F/DOG/OQ16
F/DOG/OQ16
C/CAT/MN92
C/CAT/MN92
C/DOG/MK44
C/DOG/MT12
C/DOG/MT12
C/DOG/MT12
C/DOG/MT12
C/DOG/MT12
C/DOG/MT71
C/DOG/MT71
C/DOG/OK54
C/DOG/OK54
C/DOG/OK54
C/DOG/OM64
C/DOG/OM64

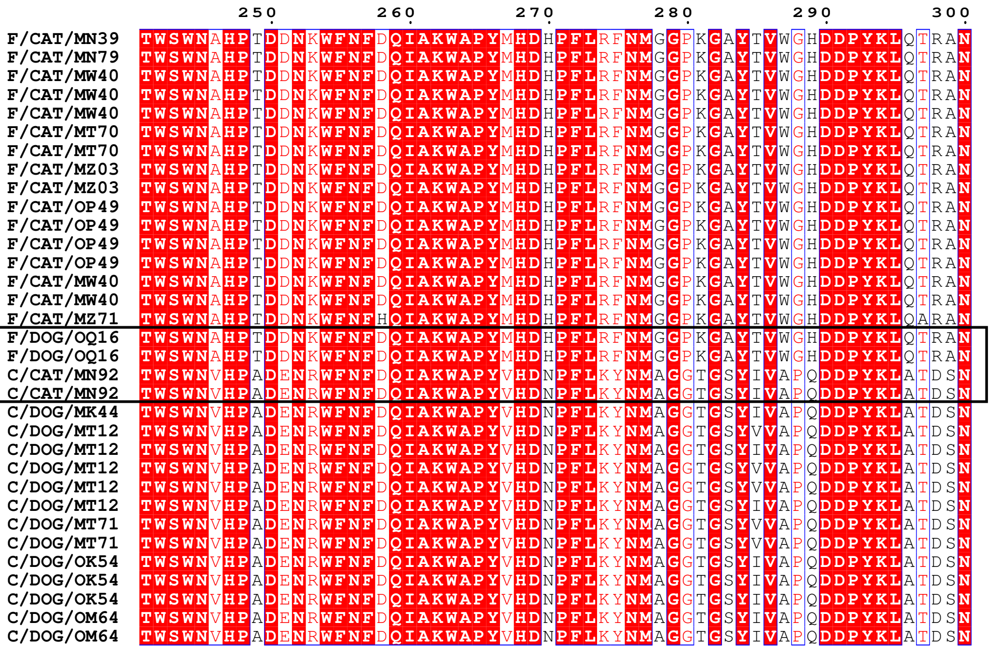
250 260 270 280 290 300
F/CAT/MN39 TWSWNAHPTDDNKWFNFDQIAKWAPYMHDHPFLRFNMGGPKGAYTVWGHDDPYKLQTRAN
F/CAT/MN79 TWSWNAHPTDDNKWFNFDQIAKWAPYMHDHPFLRFNMGGPKGAYTVWGHDDPYKLQTRAN
F/CAT/MW40 TWSWNAHPTDDNKWFNFDQIAKWAPYMHDHPFLRFNMGGPKGAYTVWGHDDPYKLQTRAN
F/CAT/MW40 TWSWNAHPTDDNKWFNFDQIAKWAPYMHDHPFLRFNMGGPKGAYTVWGHDDPYKLQTRAN
F/CAT/MW40 TWSWNAHPTDDNKWFNFDQIAKWAPYMHDHPFLRFNMGGPKGAYTVWGHDDPYKLQTRAN
F/CAT/MT70 TWSWNAHPTDDNKWFNFDQIAKWAPYMHDHPFLRFNMGGPKGAYTVWGHDDPYKLQTRAN
F/CAT/MT70 TWSWNAHPTDDNKWFNFDQIAKWAPYMHDHPFLRFNMGGPKGAYTVWGHDDPYKLQTRAN
F/CAT/MZ03 TWSWNAHPTDDNKWFNFDQIAKWAPYMHDHPFLRFNMGGPKGAYTVWGHDDPYKLQTRAN
F/CAT/MZ03 TWSWNAHPTDDNKWFNFDQIAKWAPYMHDHPFLRFNMGGPKGAYTVWGHDDPYKLQTRAN
F/CAT/OP49 TWSWNAHPTDDNKWFNFDQIAKWAPYMHDHPFLRFNMGGPKGAYTVWGHDDPYKLQTRAN
F/CAT/OP49 TWSWNAHPTDDNKWFNFDQIAKWAPYMHDHPFLRFNMGGPKGAYTVWGHDDPYKLQTRAN
F/CAT/OP49 TWSWNAHPTDDNKWFNFDQIAKWAPYMHDHPFLRFNMGGPKGAYTVWGHDDPYKLQTRAN
F/CAT/OP49 TWSWNAHPTDDNKWFNFDQIAKWAPYMHDHPFLRFNMGGPKGAYTVWGHDDPYKLQTRAN
F/CAT/MW40 TWSWNAHPTDDNKWFNFDQIAKWAPYMHDHPFLRFNMGGPKGAYTVWGHDDPYKLQTRAN
F/CAT/MW40 TWSWNAHPTDDNKWFNFDQIAKWAPYMHDHPFLRFNMGGPKGAYTVWGHDDPYKLQTRAN
F/CAT/MZ71 TWSWNAHPTDDNKWFNFHQIAKWAPYMHDHPFLRFNMGGPKGAYTVWGHDDPYKLQARAN
F/DOG/OQ16 TWSWNAHPTDDNKWFNFDQIAKWAPYMHDHPFLRFNMGGPKGAYTVWGHDDPYKLQTRAN
F/DOG/OQ16 TWSWNAHPTDDNKWFNFDQIAKWAPYMHDHPFLRFNMGGPKGAYTVWGHDDPYKLQTRAN
C/CAT/MN92 TWSWNVHPADENRWFNFDQIAKWAPYVHDNPFLKYNMAGGTGSYIVAPQDDPYKLATDSN
C/CAT/MN92 TWSWNVHPADENRWFNFDQIAKWAPYVHDNPFLKYNMAGGTGSYIVAPQDDPYKLATDSN
C/DOG/MK44 TWSWNVHPADENRWFNFDQIAKWAPYVHDNPFLKYNMAGGTGSYIVAPQDDPYKLATDSN
C/DOG/MT12 TWSWNVHPADENRWFNFDQIAKWAPYVHDNPFLKYNMAGGTGSYVVAPQDDPYKLATDSN
C/DOG/MT12 TWSWNVHPADENRWFNFDQIAKWAPYVHDNPFLKYNMAGGTGSYIVAPQDDPYKLATDSN
C/DOG/MT12 TWSWNVHPADENRWFNFDQIAKWAPYVHDNPFLKYNMAGGTGSYVVAPQDDPYKLATDSN
C/DOG/MT12 TWSWNVHPADENRWFNFDQIAKWAPYVHDNPFLKYNMAGGTGSYVVAPQDDPYKLATDSN
C/DOG/MT12 TWSWNVHPADENRWFNFDQIAKWAPYVHDNPFLKYNMAGGTGSYIVAPQDDPYKLATDSN
C/DOG/MT71 TWSWNVHPADENRWFNFDQIAKWAPYVHDNPFLKYNMAGGTGSYVVAPQDDPYKLATDSN
C/DOG/MT71 TWSWNVHPADENRWFNFDQIAKWAPYVHDNPFLKYNMAGGTGSYVVAPQDDPYKLATDSN
C/DOG/OK54 TWSWNVHPADENRWFNFDQIAKWAPYVHDNPFLKYNMAGGTGSYIVAPQDDPYKLATDSN
C/DOG/OK54 TWSWNVHPADENRWFNFDQIAKWAPYVHDNPFLKYNMAGGTGSYIVAPQDDPYKLATDSN
C/DOG/OK54 TWSWNVHPADENRWFNFDQIAKWAPYVHDNPFLKYNMAGGTGSYIVAPQDDPYKLATDSN
C/DOG/OM64 TWSWNVHPADENRWFNFDQIAKWAPYVHDNPFLKYNMAGGTGSYIVAPQDDPYKLATDSN
C/DOG/OM64 TWSWNVHPADENRWFNFDQIAKWAPYVHDNPFLKYNMAGGTGSYIVAPQDDPYKLATDSN

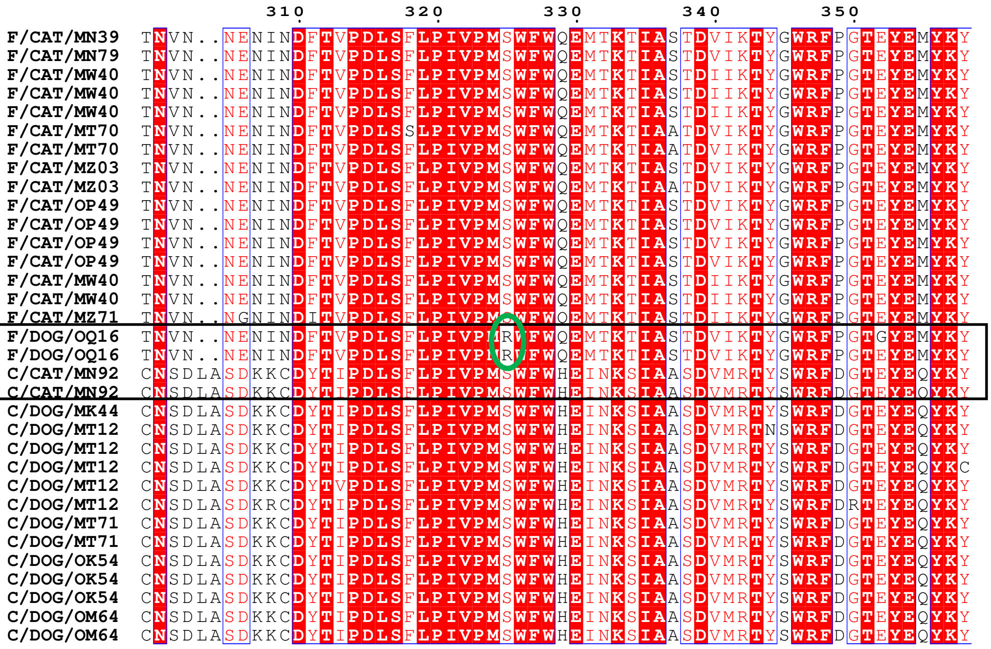
310 320 330 340 350
F/CAT/MN39 TNVN..NENINDFTVPDLSFLPIVPMSWFWQEMTKTIASTDVIKTYGWRFPGTEYEMYKY
F/CAT/MN79 TNVN..NENINDFTVPDLSFLPIVPMSWFWQEMTKTIASTDVIKTYGWRFPGTEYEMYKY
F/CAT/MW40 TNVN..NENINDFTVPDLSFLPIVPMSWFWQEMTKTIASTDIIKTYGWRFPGTEYEMYKY
F/CAT/MW40 TNVN..NENINDFTVPDLSFLPIVPMSWFWQEMTKTIASTDIIKTYGWRFPGTEYEMYKY
F/CAT/MW40 TNVN..NENINDFTVPDLSFLPIVPMSWFWQEMTKTIASTDIIKTYGWRFPGTEYEMYKY
F/CAT/MT70 TNVN..NENINDFTVPDLSSLPIVPMSWFWQEMTKTIAATDVIKTYGWRFPGTEYEMYKY
F/CAT/MT70 TNVN..NENINDFTVPDLSFLPIVPMSWFWQEMTKTIAATDVIKTYGWRFPGTEYEMYKY
F/CAT/MZ03 TNVN..NENINDFTVPDLSFLPIVPMSWFWQEMTKTIASTDVIKTYGWRFPGTEYEMYKY
F/CAT/MZ03 TNVN..NENINDFTVPDLSFLPIVPMSWFWQEMTKTIAATDVIKTYGWRFPGTEYEMYKY
F/CAT/OP49 TNVN..NENINDFTVPDLSFLPIVPMSWFWQEMTKTIASTDVIKTYGWRFPGTEYEMYKY
F/CAT/OP49 TNVN..NENINDFTVPDLSFLPIVPMSWFWQEMTKTIASTDVIKTYGWRFPGTEYEMYKY
F/CAT/OP49 TNVN..NENINDFTVPDLSFLPIVPMSWFWQEMTKTIASTDVIKTYGWRFPGTEYEMYKY
F/CAT/OP49 TNVN..NENINDFTVPDLSFLPIVPMSWFWQEMTKTIASTDVIKTYGWRFPGTEYEMYKY
F/CAT/MW40 TNVN..NENINDFTVPDLSFLPIVPMSWFWQEMTKTIASTDIIKTYGWRFPGTEYEMYKY
F/CAT/MW40 TNVN..NENINDFTVPDLSFLPIVPMSWFWQEMTKTIASTDIIKTYGWRFPGTEYEMYKY
F/CAT/MZ71 TNVN..NGNINDITVPDLSFLPIVPMSWFWQEMTKTIASTDIIKTYGWRFPGTEYEMYKY
F/DOG/OQ16 TNVN..NENINDFTVPDLSFLPIVPMRWFWQEMTKTIASTDVIKTYGWRFPGTGYEMYKY
F/DOG/OQ16 TNVN..NENINDFTVPDLSFLPIVPMRWFWQEMTKTIASTDVIKTYGWRFPGTEYEMYKY
C/CAT/MN92 CNSDLASDKKCDYTIPDLSFLPIVPMSWFWHEINKSIAASDVMRTYSWRFDGTEYEQYKY
C/CAT/MN92 CNSDLASDKKCDYTIPDLSFLPIVPMSWFWHEINKSIAASDVMRTYSWRFDGTEYEQYKY
C/DOG/MK44 CNSDLASDKKCDYTIPDLSFLPIVPMSWFWHEINKSIAASDVMRTYSWRFDGTEYEQYKY
C/DOG/MT12 CNSDLASDKKCDYTIPDLSFLPIVPMSWFWHEINKSIAASDVMRTNSWRFDGTEYEQYKY
C/DOG/MT12 CNSDLASDKKCDYTIPDLSFLPIVPMSWFWHEINKSIAASDVMRTYSWRFDGTEYEQYKY
C/DOG/MT12 CNSDLASDKKCDYTIPDLSFLPIVPMSWFWHEINKSIAASDVMRTYSWRFDGTEYEQYKC
C/DOG/MT12 CNSDLASDKKCDYTVPDLSFLPIVPMSWFWHEINKSIAASDVMRTYSWRFDGTEYEQYKY
C/DOG/MT12 CNSDLASDKRCDYTIPDLSFLPIVPMSWFWHEINKSIAASDVMRTYSWRFDRTEYEQYKY
C/DOG/MT71 CNSDLASDKKCDYTIPDLSFLPIVPMSWFWHEINKSIAASDVMRTYSWRFDGTEYEQYKY
C/DOG/MT71 CNSDLASDKKCDYTIPDLSFLPIVPMSWFWHEINKSIAASDVMRTYSWRFDGTEYEQYKY
C/DOG/OK54 CNSDLASDKKCDYTIPDLSFLPIVPMSWFWHEINKSIAASDVMRTYSWRFDGTEYEQYKY
C/DOG/OK54 CNSDLASDKKCDYTIPDLSFLPIVPMSWFWHEINKSIAASDVMRTYSWRFDGTEYEQYKY
C/DOG/OK54 CNSDLASDKKCDYTIPDLSFLPIVPMSWFWHEINKSIAASDVMRTYSWRFDGTEYEQYKY
C/DOG/OM64 CNSDLASDKKCDYTIPDLSFLPIVPMSWFWHEINKSIAASDVMRTYSWRFDGTEYEQYKY
C/DOG/OM64 CNSDLASDKKCDYTIPDLSFLPIVPMSWFWHEINKSIAASDVMRTYSWRFDGTEYEQYKY

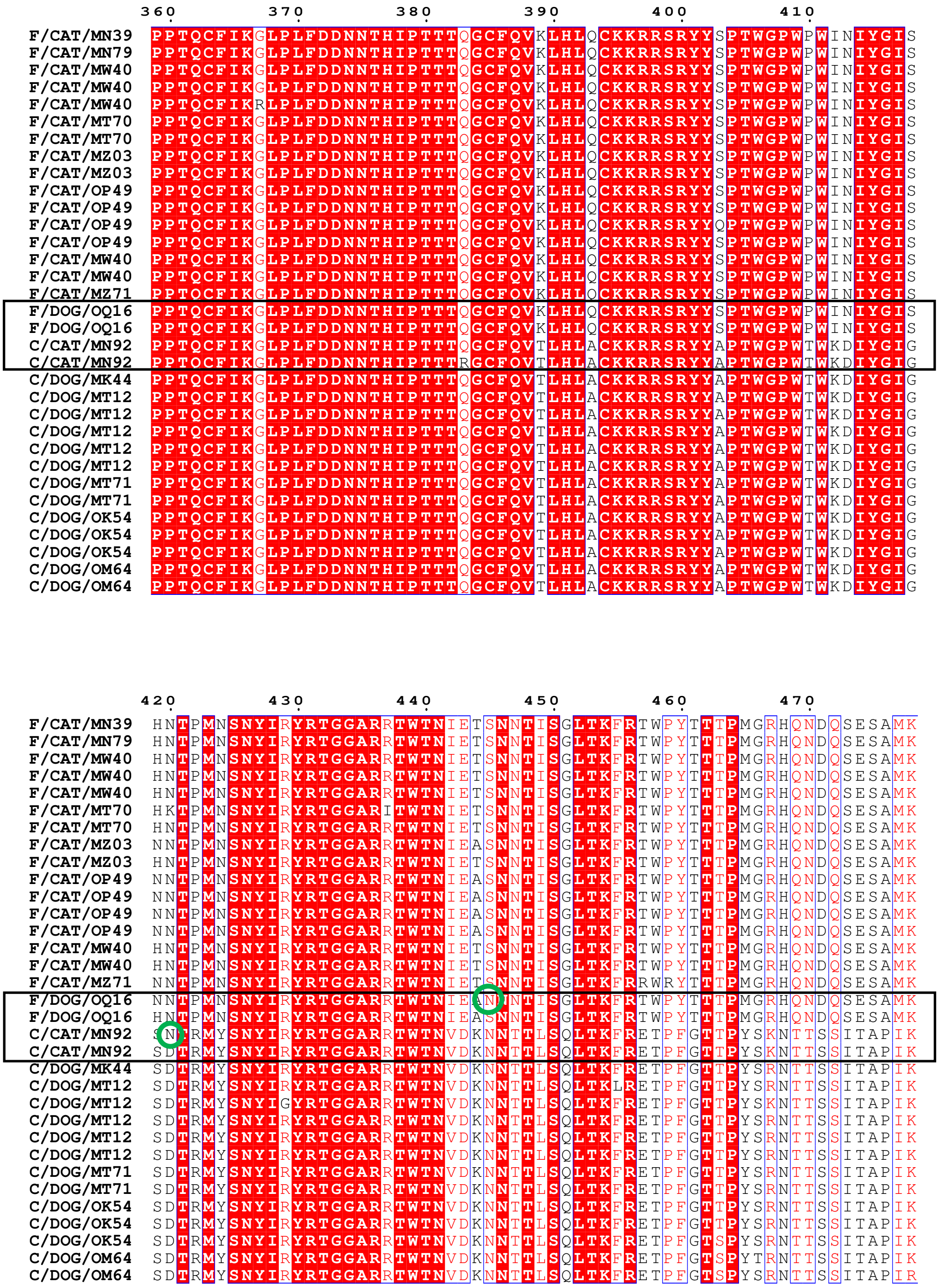
360 370 380 390 400 410
F/CAT/MN39 PPTQCFIKGLPLFDDNNTHIPTTTQGCFQVKLHLQCKKRRSRYYSPTWGPWPWINIYGIS
F/CAT/MN79 PPTQCFIKGLPLFDDNNTHIPTTTQGCFQVKLHLQCKKRRSRYYSPTWGPWPWINIYGIS
F/CAT/MW40 PPTQCFIKGLPLFDDNNTHIPTTTQGCFQVKLHLQCKKRRSRYYSPTWGPWPWINIYGIS
F/CAT/MW40 PPTQCFIKGLPLFDDNNTHIPTTTQGCFQVKLHLQCKKRRSRYYSPTWGPWPWINIYGIS
F/CAT/MW40 PPTQCFIKRLPLFDDNNTHIPTTTQGCFQVKLHLQCKKRRSRYYSPTWGPWPWINIYGIS
F/CAT/MT70 PPTQCFIKGLPLFDDNNTHIPTTTQGCFQVKLHLQCKKRRSRYYSPTWGPWPWINIYGIS
F/CAT/MT70 PPTQCFIKGLPLFDDNNTHIPTTTQGCFQVKLHLQCKKRRSRYYSPTWGPWPWINIYGIS
F/CAT/MZ03 PPTQCFIKGLPLFDDNNTHIPTTTQGCFQVKLHLQCKKRRSRYYSPTWGPWPWINIYGIS
F/CAT/MZ03 PPTQCFIKGLPLFDDNNTHIPTTTQGCFQVKLHLQCKKRRSRYYSPTWGPWPWINIYGIS
F/CAT/OP49 PPTQCFIKGLPLFDDNNTHIPTTTQGCFQVKLHLQCKKRRSRYYSPTWGPWPWINIYGIS
F/CAT/OP49 PPTQCFIKGLPLFDDNNTHIPTTTQGCFQVKLHLQCKKRRSRYYSPTWGPWPWINIYGIS
F/CAT/OP49 PPTQCFIKGLPLFDDNNTHIPTTTQGCFQVKLHLQCKKRRSRYYQPTWGPWPWINIYGIS
F/CAT/OP49 PPTQCFIKGLPLFDDNNTHIPTTTQGCFQVKLHLQCKKRRSRYYSPTWGPWPWINIYGIS
F/CAT/MW40 PPTQCFIKGLPLFDDNNTHIPTTTQGCFQVKLHLQCKKRRSRYYSPTWGPWPWINIYGIS
F/CAT/MW40 PPTQCFIKGLPLFDDNNTHIPTTTQGCFQVKLHLQCKKRRSRYYSPTWGPWPWINIYGIS
F/CAT/MZ71 PPTQCFIKGLPLFDDNNTHIPTTTQGCFQVKLHLQCKKRRSRYYSPTWGPWPWINIYGIS
F/DOG/OQ16 PPTQCFIKGLPLFDDNNTHIPTTTQGCFQVKLHLQCKKRRSRYYSPTWGPWPWINIYGIS
F/DOG/OQ16 PPTQCFIKGLPLFDDNNTHIPTTTQGCFQVKLHLQCKKRRSRYYSPTWGPWPWINIYGIS
C/CAT/MN92 PPTQCFIKGLPLFDDNNTHIPTTTQGCFQVTLHLACKKRRSRYYAPTWGPWTWKDIYGIG
C/CAT/MN92 PPTQCFIKGLPLFDDNNTHIPTTTRGCFQVTLHLACKKRRSRYYAPTWGPWTWKDIYGIG
C/DOG/MK44 PPTQCFIKGLPLFDDNNTHIPTTTQGCFQVTLHLACKKRRSRYYAPTWGPWTWKDIYGIG
C/DOG/MT12 PPTQCFIKGLPLFDDNNTHIPTTTQGCFQVTLHLACKKRRSRYYAPTWGPWTWKDIYGIG
C/DOG/MT12 PPTQCFIKGLPLFDDNNTHIPTTTQGCFQVTLHLACKKRRSRYYAPTWGPWTWKDIYGIG
C/DOG/MT12 PPTQCFIKGLPLFDDNNTHIPTTTQGCFQVTLHLACKKRRSRYYAPTWGPWTWKDIYGIG
C/DOG/MT12 PPTQCFIKGLPLFDDNNTHIPTTTQGCFQVTLHLACKKRRSRYYAPTWGPWTWKDIYGIG
C/DOG/MT12 PPTQCFIKGLPLFDDNNTHIPTTTQGCFQVTLHLACKKRRSRYYAPTWGPWTWKDIYGIG
C/DOG/MT71 PPTQCFIKGLPLFDDNNTHIPTTTQGCFQVTLHLACKKRRSRYYAPTWGPWTWKDIYGIG
C/DOG/MT71 PPTQCFIKGLPLFDDNNTHIPTTTQGCFQVTLHLACKKRRSRYYAPTWGPWTWKDIYGIG
C/DOG/OK54 PPTQCFIKGLPLFDDNNTHIPTTTQGCFQVTLHLACKKRRSRYYAPTWGPWTWKDIYGIG
C/DOG/OK54 PPTQCFIKGLPLFDDNNTHIPTTTQGCFQVTLHLACKKRRSRYYAPTWGPWTWKDIYGIG
C/DOG/OK54 PPTQCFIKGLPLFDDNNTHIPTTTQGCFQVTLHLACKKRRSRYYAPTWGPWTWKDIYGIG
C/DOG/OM64 PPTQCFIKGLPLFDDNNTHIPTTTQGCFQVTLHLACKKRRSRYYAPTWGPWTWKDIYGIG
C/DOG/OM64 PPTQCFIKGLPLFDDNNTHIPTTTQGCFQVTLHLACKKRRSRYYAPTWGPWTWKDIYGIG
420 430 440 450 460 470
F/CAT/MN39 HNTPMNSNYIRYRTGGARRTWTNIETSNNTISGLTKFRTWPYTTTPMGRHQNDQSESAMK
F/CAT/MN79 HNTPMNSNYIRYRTGGARRTWTNIETSNNTISGLTKFRTWPYTTTPMGRHQNDQSESAMK
F/CAT/MW40 HNTPMNSNYIRYRTGGARRTWTNIETSNNTISGLTKFRTWPYTTTPMGRHQNDQSESAMK
F/CAT/MW40 HNTPMNSNYIRYRTGGARRTWTNIETSNNTISGLTKFRTWPYTTTPMGRHQNDQSESAMK
F/CAT/MW40 HNTPMNSNYIRYRTGGARRTWTNIETSNNTISGLTKFRTWPYTTTPMGRHQNDQSESAMK
F/CAT/MT70 HKTPMNSNYIRYRTGGARITWTNIETSNNTISGLTKFRTWPYTTTPMGRHQNDQSESAMK
F/CAT/MT70 HNTPMNSNYIRYRTGGARRTWTNIETSNNTISGLTKFRTWPYTTTPMGRHQNDQSESAMK
F/CAT/MZ03 NNTPMNSNYIRYRTGGARRTWTNIEASNNTISGLTKFRTWPYTTTPMGRHQNDQSESAMK
F/CAT/MZ03 HNTPMNSNYIRYRTGGARRTWTNIETSNNTISGLTKFRTWPYTTTPMGRHQNDQSESAMK
F/CAT/OP49 NNTPMNSNYIRYRTGGARRTWTNIEASNNTISGLTKFRTWPYTTTPMGRHQNDQSESAMK
F/CAT/OP49 NNTPMNSNYIRYRTGGARRTWTNIEASNNTISGLTKFRTWPYTTTPMGRHQNDQSESAMK
F/CAT/OP49 NNTPMNSNYIRYRTGGARRTWTNIEASNNTISGLTKFRTWPYTTTPMGRHQNDQSESAMK
F/CAT/OP49 NNTPMNSNYIRYRTGGARRTWTNIEASNNTISGLTKFRTWPYTTTPMGRHQNDQSESAMK
F/CAT/MW40 HNTPMNSNYIRYRTGGARRTWTNIETSNNTISGLTKFRTWPYTTTPMGRHQNDQSESAMK
F/CAT/MW40 HNTPMNSNYIRYRTGGARRTWTNIETSNNTISGLTKFRTWPYTTTPMGRHQNDQSESAMK
F/CAT/MZ71 NNTPMNSNYIRYRTGGARRTWTNIETSNNTISGLTKFRRWRYTTTPMGRHQNDQSESAMK
F/DOG/OQ16 NNTPMNSNYIRYRTGGARRTWTNIEANNNTISGLTKFRTWPYTTTPMGRHQNDQSESAMK
F/DOG/OQ16 HNTPMNSNYIRYRTGGARRTWTNIEASNNTISGLTKFRTWPYTTTPMGRHQNDQSESAMK
C/CAT/MN92 SNTRMYSNYIRYRTGGARRTWTNVDKNNTTLSQLTKFRETPFGTTPYSKNTTSSITAPIK
C/CAT/MN92 SDTRMYSNYIRYRTGGARRTWTNVDKNNTTLSQLTKFRETPFGTTPYSKNTTSSITAPIK
C/DOG/MK44 SDTRMYSNYIRYRTGGARRTWTNVDKNNTTLSQLTKFRETPFGTTPYSRNTTSSITAPIK
C/DOG/MT12 SDTRMYSNYIRYRTGGARRTWTNVDKNNTTLSQLTKLRETPFGTTPYSRNTTSSITAPIK
C/DOG/MT12 SDTRMYSNYIGYRTGGARRTWTNVDKNNTTLSQLTKFRETPFGTTPYSKNTTSSITAPIK
C/DOG/MT12 SDTRMYSNYIRYRTGGARRTWTNVDKNNTTLSQLTKFRETPFGTTPYSRNTTSSITAPIK
C/DOG/MT12 SDTRMYSNYIRYRTGGARRTWTNVDKNNTTLSQLTKFRETPFGTTPYSRNTTSSITAPIK
C/DOG/MT12 SDTRMYSNYIRYRTGGARRTWTNVDKNNTTLSQLTKFRETPFGTTPYSRNTTSSITAPIK
C/DOG/MT71 SDTRMYSNYIRYRTGGARRTWTNVDKNNTTLSQLTKFRETPFGTTPYSRNTTSSITAPIK
C/DOG/MT71 SDTRMYSNYIRYRTGGARRTWTNVDKNNTTLSQLTKFRETPFGTTPYSRNTTSSITAPIK
C/DOG/OK54 SDTRMYSNYIRYRTGGARRTWTNVDKNNTTLSQLTKFRETPFGTTPYSRNTTSSITAPIK
C/DOG/OK54 SDTRMYSNYIRYRTGGARRTWTNVDKNNTTLSQLTKFRETPFGTTPYSRNTTSSITAPIK
C/DOG/OK54 SDTRMYSNYIRYRTGGARRTWTNVDKNNTTLSQLTKFRETPFGTSPYSRNTTSSITAPIK
C/DOG/OM64 SDTRMYSNYIRYRTGGARRTWTNVDKNNTTLSQLTKFRETPFGTSPYTRNTTSSITAPIK
C/DOG/OM64 SDTRMYSNYIRYRTGGARRTWTNVDKNNTTLSQLTKFRETPFGTSPYSRNTTSSITAPIR

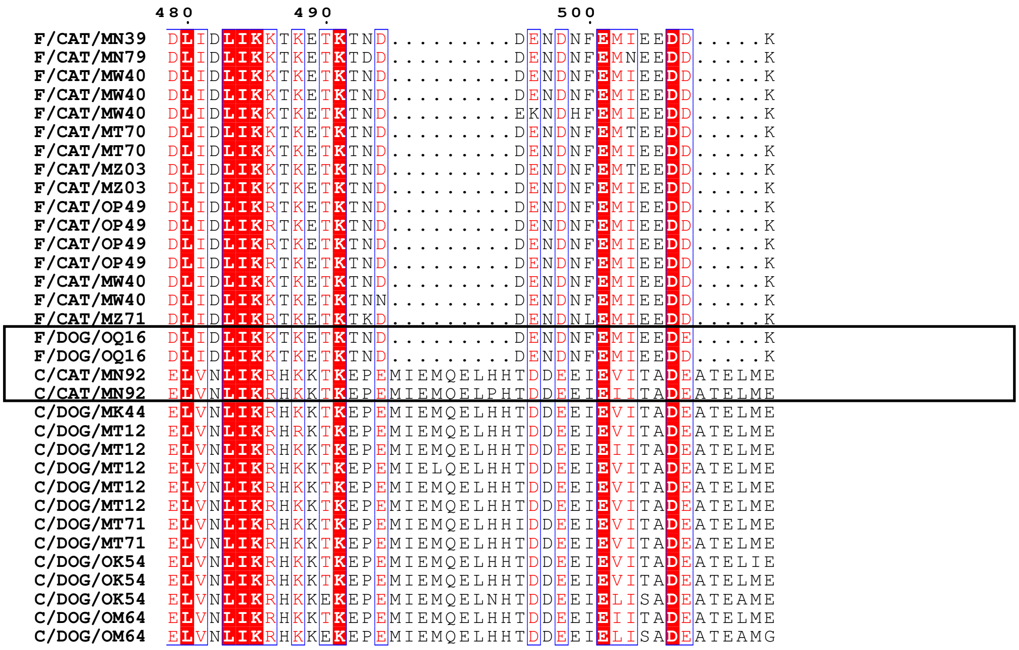
480
490
500
F/CAT/MN39 DLIDLIKKTKETKTND.........DENDNFEMIEEDD.....K
F/CAT/MN79 DLIDLIKKTKETKTDD.........DENDNFEMNEEDD.....K
F/CAT/MW40 DLIDLIKKTKETKTND.........DENDNFEMIEEDD.....K
F/CAT/MW40 DLIDLIKKTKETKTND.........DENDNFEMIEEDD.....K
F/CAT/MW40 DLIDLIKKTKETKTND.........EKNDHFEMIEEDD.....K
F/CAT/MT70 DLIDLIKKTKETKTND.........DENDNFEMTEEDD.....K
F/CAT/MT70 DLIDLIKKTKETKTND.........DENDNFEMIEEDD.....K
F/CAT/MZ03 DLIDLIKKTKETKTND.........DENDNFEMTEEDD.....K
F/CAT/MZ03 DLIDLIKKTKETKTND.........DENDNFEMIEEDD.....K
F/CAT/OP49 DLIDLIKRTKETKTND.........DENDNFEMIEEDD.....K
F/CAT/OP49 DLIDLIKRTKETKTND.........DENDNFEMIEEDD.....K
F/CAT/OP49 DLIDLIKRTKETKTND.........DENDNFEMIEEDD.....K
F/CAT/OP49 DLIDLIKRTKETKTND.........DENDNFEMIEEDD.....K
F/CAT/MW40 DLIDLIKKTKETKTND.........DENDNFEMIEEDD.....K
F/CAT/MW40 DLIDLIKKTKETKTNN.........DENDNFEMIEEDD.....K
F/CAT/MZ71 DLIDLIKRTKETKTKD.........DENDNLEMIEEDD.....K
F/DOG/OQ16 DLIDLIKKTKETKTND.........DENDNFEMIEEDE.....K
F/DOG/OQ16 DLIDLIKKTKETKTND.........DENDNFEMIEEDD.....K
C/CAT/MN92 ELVNLIKRHKKTKEPEMIEMQELHHTDDEEIEVITADEATELME
C/CAT/MN92 ELVNLIKRHKKTKEPEMIEMQELPHTDDEEIEIITADEATELME
C/DOG/MK44 ELVNLIKRHKKTKEPEMIEMQELHHTDDEEIEVITADEATELME
C/DOG/MT12 ELVNLIKRHRKTKEPEMIEMQELHHTDDEEIEVITADEATELME
C/DOG/MT12 ELVNLIKRHKKTKEPEMIEMQELHHTDDEEIEIITADEATELME
C/DOG/MT12 ELVNLIKRHKKTKEPEMIELQELHHTDDEEIEVITADEATELME
C/DOG/MT12 ELVNLIKRHKKTKEPEMIEMQELHHTDDEEIEVITADEATELME
C/DOG/MT12 ELVNLIKRHKKTKEPEMIEMQELHHTDDEEIEVITADEATELME
C/DOG/MT71 ELVNLIKRHKKTKEPEMIEMQELHHIDDEEIEVITADEATELME
C/DOG/MT71 ELVNLIKRHKKTKEPEMIEMQELHHTDDEEIEVITADEATELME
C/DOG/OK54 ELVNLIKRHKKTKEPEMIEMQELHHTDDEEIEVITADEATELIE
C/DOG/OK54 ELVNLIKRHKKTKEPEMIEMQELHHTDDEEIEVITADEATELME
C/DOG/OK54 ELVNLIKRHKKEKEPEMIEMQELNHTDDEEIELISADEATEAME
C/DOG/OM64 ELVNLIKRHKKTKEPEMIEMQELHHTDDEEIEIITADEATELME
C/DOG/OM64 ELVNLIKRHKKEKEPEMIEMQELHHTDDEEIELISADEATEAMG
